# SHock-INduced Endotheliopathy (SHINE): A mechanistic justification for viscoelastography-guided resuscitation of traumatic and non-traumatic shock

**DOI:** 10.3389/fphys.2023.1094845

**Published:** 2023-02-27

**Authors:** Connor M. Bunch, Eric Chang, Ernest E. Moore, Hunter B. Moore, Hau C. Kwaan, Joseph B. Miller, Mahmoud D. Al-Fadhl, Anthony V. Thomas, Nuha Zackariya, Shivani S. Patel, Sufyan Zackariya, Saadeddine Haidar, Bhavesh Patel, Michael T. McCurdy, Scott G. Thomas, Donald Zimmer, Daniel Fulkerson, Paul Y. Kim, Matthew R. Walsh, Daniel Hake, Archana Kedar, Michael Aboukhaled, Mark M. Walsh

**Affiliations:** ^1^ Department of Emergency Medicine, Henry Ford Hospital, Detroit, MI, United States; ^2^ Department of Internal Medicine, Henry Ford Hospital, Detroit, MI, United States; ^3^ Department of Medical Education, Indiana University School of Medicine, Notre Dame Campus, South Bend, IN, United States; ^4^ Department of Surgery, Ernest E. Moore Shock Trauma Center at Denver Health, University of Colorado, Denver, CO, United States; ^5^ Department of Transplant Surgery, Denver Health and University of Colorado Health Sciences Center, Denver, CO, United States; ^6^ Division of Hematology and Oncology, Department of Medicine, Northwestern University Feinberg School of Medicine, Chicago, IL, United States; ^7^ Division of Critical Care, Department of Medicine, Mayo Clinic Arizona, Phoenix, AZ, United States; ^8^ Division of Pulmonary and Critical Care, Department of Medicine, University of Maryland School of Medicine, Baltimore, MD, United States; ^9^ Department of Trauma Surgery, Memorial Leighton Trauma Center, South Bend, IN, United States; ^10^ Department of Medicine, McMaster University, Hamilton, ON, Canada; ^11^ Thrombosis and Atherosclerosis Research Institute, Hamilton, ON, Canada; ^12^ Cardinal Flow Assurance LLC, Mishawaka, IN, United States; ^13^ Departments of Emergency Medicine and Internal Medicine, Saint Joseph Regional Medical Center, Mishawaka, IN, United States

**Keywords:** critical care, endothelium, glycocalyx, hemostasis, precision medicine, resuscitation, shock, thromboelastography

## Abstract

Irrespective of the reason for hypoperfusion, hypocoagulable and/or hyperfibrinolytic hemostatic aberrancies afflict up to one-quarter of critically ill patients in shock. Intensivists and traumatologists have embraced the concept of SHock-INduced Endotheliopathy (SHINE) as a foundational derangement in progressive shock wherein sympatho-adrenal activation may cause systemic endothelial injury. The pro-thrombotic endothelium lends to micro-thrombosis, enacting a cycle of worsening perfusion and increasing catecholamines, endothelial injury, de-endothelialization, and multiple organ failure. The hypocoagulable/hyperfibrinolytic hemostatic phenotype is thought to be driven by endothelial release of anti-thrombogenic mediators to the bloodstream and perivascular sympathetic nerve release of tissue plasminogen activator directly into the microvasculature. In the shock state, this hemostatic phenotype may be a counterbalancing, yet maladaptive, attempt to restore blood flow against a systemically pro-thrombotic endothelium and increased blood viscosity. We therefore review endothelial physiology with emphasis on glycocalyx function, unique biomarkers, and coagulofibrinolytic mediators, setting the stage for understanding the pathophysiology and hemostatic phenotypes of SHINE in various etiologies of shock. We propose that the hyperfibrinolytic phenotype is exemplified in progressive shock whether related to trauma-induced coagulopathy, sepsis-induced coagulopathy, or post-cardiac arrest syndrome-associated coagulopathy. Regardless of the initial insult, SHINE appears to be a catecholamine-driven entity which early in the disease course may manifest as hyper- or hypocoagulopathic and hyper- or hypofibrinolytic hemostatic imbalance. Moreover, these hemostatic derangements may rapidly evolve along the thrombohemorrhagic spectrum depending on the etiology, timing, and methods of resuscitation. Given the intricate hemochemical makeup and changes during these shock states, macroscopic whole blood tests of coagulative kinetics and clot strength serve as clinically useful and simple means for hemostasis phenotyping. We suggest that viscoelastic hemostatic assays such as thromboelastography (TEG) and rotational thromboelastometry (ROTEM) are currently the most applicable clinical tools for assaying global hemostatic function—including fibrinolysis—to enable dynamic resuscitation with blood products and hemostatic adjuncts for those patients with thrombotic and/or hemorrhagic complications in shock states.

## 1 Introduction: “The machinery of life has been rudely unhinged”

Of the many descriptions of shock, the first by Samuel Gross in the year 1882 as when “the machinery of life has been rudely unhinged” remains concise and accurate (Millham, 2010). This narrative review expands upon the classic descriptions of shock to elucidate the unifying potential for SHock-INduced Endotheliopathy (SHINE) to explain the hemostatic aberrancies observed in all etiologies of progressive shock. Approximately one-quarter of severely injured trauma patients develop hemostatic derangement with an associated increased mortality of three to four times higher than those without coagulopathy. Increased mortality is also noted for critically ill patients in shock with coagulopathies not caused by trauma (Brohi et al., 2003; Adrie et al., 2004; Hess et al., 2008; Johansson et al., 2011a; Gando et al., 2011; Holcomb et al., 2012; Angus and Van der Poll, 2013; Kim et al., 2013; Wada, 2017; Bugaev et al., 2020; Walsh et al., 2020b; Iba and Levy, 2020; Moore et al., 2020). Due to its ubiquitous distribution, substantial surface area, and interfacing role in hemostasis, immunology, and blood flow, the endothelium serves as a common foundation for hemostatic derangement associated with all forms of shock (Fishman, 1982; Aird, 2004; Johansson et al., 2017a).

Here, the physiologic roles of the endothelium are detailed with particular emphasis on coagulofibrinolytic balance, glycocalyx function, and unique biomarkers. Understanding these endothelial functions offers insight to the pathophysiologic anomalies at the level of the endothelium in various forms of shock. Throughout this review, the viscoelastic hemostatic assays (VHAs) thromboelastography (TEG) and rotational thromboelastometry (ROTEM) receive particular attention for their ability to globally assay coagulofibrinolysis, thus enabling a precision-based medicine (PBM) approach to diagnosing and treating the spectrum of coagulopathies associated with SHINE (Stettler et al., 2019). First, we briefly review the classic anatomic and pathophysiologic definitions of shock.

### 1.1 Epidemiology and classic categorization of shock

Among the etiologies of shock, septic and cardiogenic shock are the most common with an incidence of 171 per 100,000 population and 51.7 per 100,000 population, respectively (Dupuis et al., 2020; Schrage et al., 2021). While the incidence of septic and cardiogenic shock increases annually, the in-hospital mortality rate is slowly decreasing but remains high at 34% and 37%, respectively (Paoli et al., 2018; Osman et al., 2021).

**FIGURE 1:**
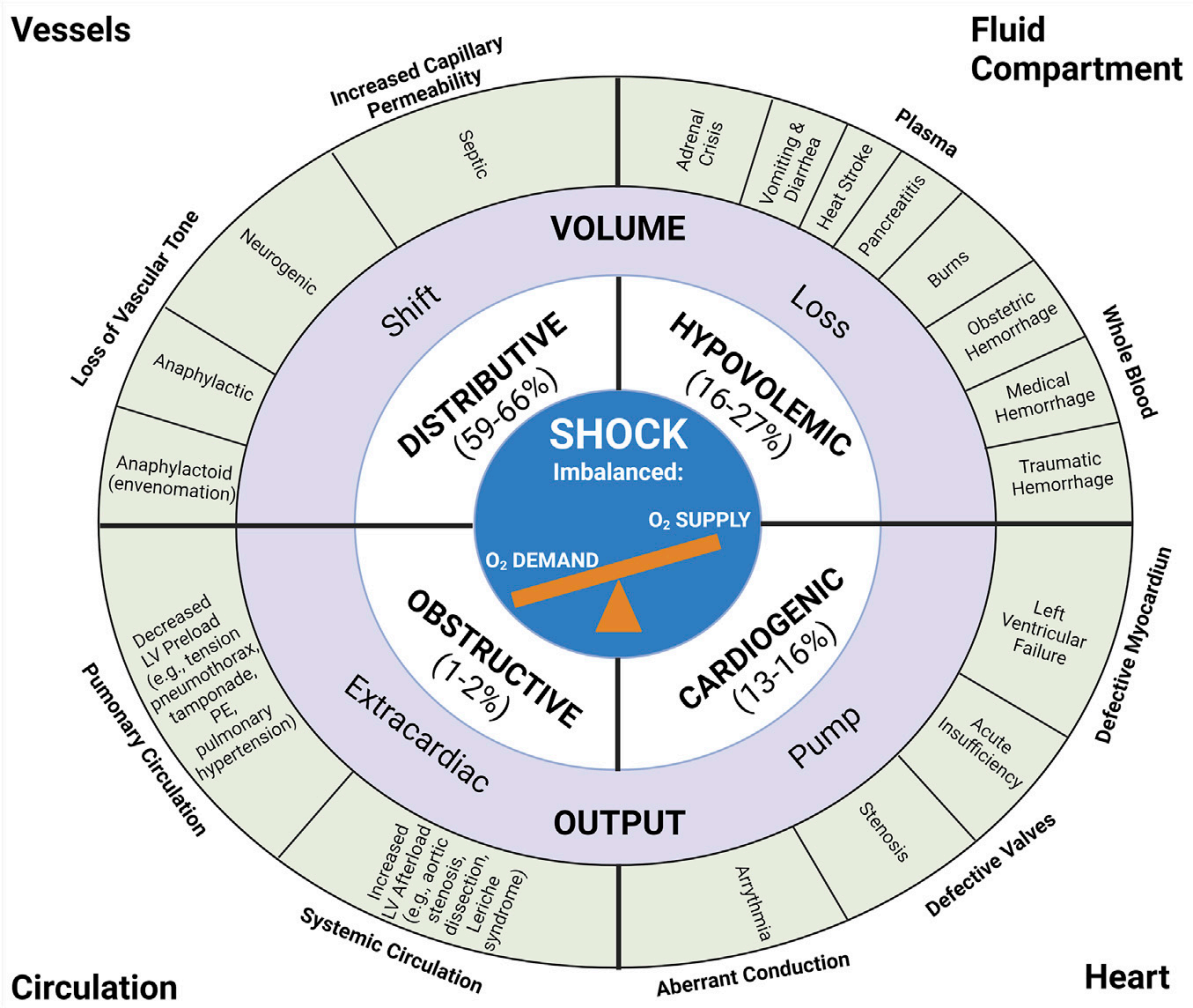
Anatomic and Pathophysiologic Categorization of Shock with Relative Frequency of Each Type. The traditional classification of shock includes four main categories: distributive, hypovolemic, obstructive, and cardiogenic shock. The estimated relative frequency of each of the four subcategories are listed with pathophysiologic etiologies defined around the periphery of the diagram (Vincent and De Backer, 2013; Standl et al., 2018). Recreated with permission from (Standl et al., 2018). Created with BioRender.com.

Shock may be diversely defined, all definitions of which approximate the imbalance between tissue oxygen supply and demand (Millham, 2010; Johansson et al., 2017a; Standl et al., 2018). Shock has traditionally been defined by four categories based on fluid compartment volume loss (hypovolemic), volume redistribution (distributive), cardiac pump activity (cardiogenic), and circulatory obstruction (obstructive). These four etiologies of shock are summarized in Figure 1. Briefly, distributive shock pertains to the redistribution of fluid out of the intravascular space and/or away from vital organs without blood or fluid loss. Distributive shock, particularly septic shock, accounts for 59%–66% of all shock presentations (Vincent and De Backer, 2013; Standl et al., 2018). There are three subtypes of distributive shock which include septic, neurogenic, and anaphylactic/anaphylactoid (Standl et al., 2018). Primary endothelial dysfunction (as a root cause of shock) may be a key element of distributive shock and as such, primary endotheliopathy in this type of shock may be further amplified by SHINE. Hypovolemic shock results from a loss of blood or plasma and comprises an estimated 16%–27% of patients in shock (Vincent and De Backer, 2013; Standl et al., 2018). Hypovolemic shock includes four subtypes including hemorrhagic shock, traumatic hemorrhagic shock, plasma loss (non-hemorrhagic) hypovolemic shock, and traumatic hypovolemic shock (Standl et al., 2018). Whole blood loss occurs in hemorrhagic states such as trauma, gastrointestinal bleeds, and obstetrical hemorrhage. Plasma loss occurs with dehydrating conditions such as burns, pancreatitis, and diarrhea. Cardiogenic shock arises from primary pump failure of the heart and accounts for an estimated 13%–16% of shock states (Vincent and De Backer, 2013; Standl et al., 2018). Heart conditions that commonly account for cardiogenic shock include acute myocardial infraction, heart failure, arrythmia, and defective valves. Obstructive shock arises from a blockage of the circulation and accounts for 1%–2% of shock presentations (Vincent and De Backer, 2013; Standl et al., 2018). The defining treatment for obstructive shock is to find the source of obstruction (e.g., cardiac tamponade, tension pneumothorax, or pulmonary embolism) and relieve it.

### 1.2 Limitation of the anatomic and pathophysiologic classification system

This classification precludes a unified approach towards the spectrum of coagulopathies associated with shock. As opposed to the classic anatomic and pathophysiologic definitions, shock may also be described at the level of the endothelium (Fishman, 1982; Aird, 2004). The shared endothelial dysfunction in shock has recently been termed SHINE (Johansson et al., 2017a), whereby the endothelium is acknowledged as an independent “organ” which requires resuscitation in severe injury or disease. SHINE is a catecholamine-driven entity, regardless of the initial anatomic or pathophysiologic insult, which early in the disease course may manifest as hyper- or hypocoagulopathic and hyper- or hypofibrinolytic hemostatic imbalance. Moreover, these hemostatic derangements may rapidly evolve into phenotypes along the thrombohemorrhagic spectrum depending on the etiology, timing, and methods of resuscitation (Johansson et al., 2017a; Wei et al., 2018; Napolitano, 2021; Kregel et al., 2022).

### 1.3 Physiology of the endothelium

The definition of an organ requires a collection of tissues that form a unit distinct in form and function (Aird, 2004). Endothelial cells uniquely function as stress-sensing, phenotype-switching cells that respond to flow. The endothelium forms an extensive network with a collective weight of around 1 kg. In the brain alone, microvasculature represents 3%–4% of the brain compartment with a cumulative length of 400 miles and a surface area of exchange of 20 m^2^ between the brain parenchyma and blood (Pardridge, 2002; Deracinois et al., 2015; Goncharov et al., 2017). The endothelium is comprised of a luminal glycocalyx layer maintained by simple squamous endothelial cells held together by complex transmembrane and cytoskeletal components which form intercellular junctions (Reiterer and Branco, 2020). The endothelium has distinct biochemical markers including E-selectin, intercellular adhesion molecule-1 (ICAM-1), the syndecan (Syn) family of four (Syn1-Syn4) heparan sulfate proteoglycans (HSPGs), and angiopoietin (Agpt)-1 and Agpt-2 (Goncharov et al., 2017). The endothelial luminal layer also contains anti-thrombogenic factors such as antithrombin III (AT), thrombomodulin (TM), tissue factor pathway inhibitor (TFPI), and endogenous heparan sulfates (HS) (Reitsma et al., 2007). Far from an inert layer of cells lining all blood and lymphatic vessels, the endothelium plays a vital role in moderating Starling Forces, mounting or attenuating an immune response, modulating vascular resistance, angiogenesis, and regulating coagulofibrinolysis. This is all facilitated by the rapid physical (shear; pressure; contractions/dilations to maintain vascular tone), chemical (manufacture and release of coagulofibrinolytic agents, also in response to physical and electrical stimuli; control of vascular surface/adhesive chemistry and morphology) and electrical (cell activation *via* central/peripheral nervous system; intercellular communication and response *via* chemical/ionic configurations) feedback mechanisms central to the endothelium’s role in maintaining hemostasis.

**FIGURE 2:**
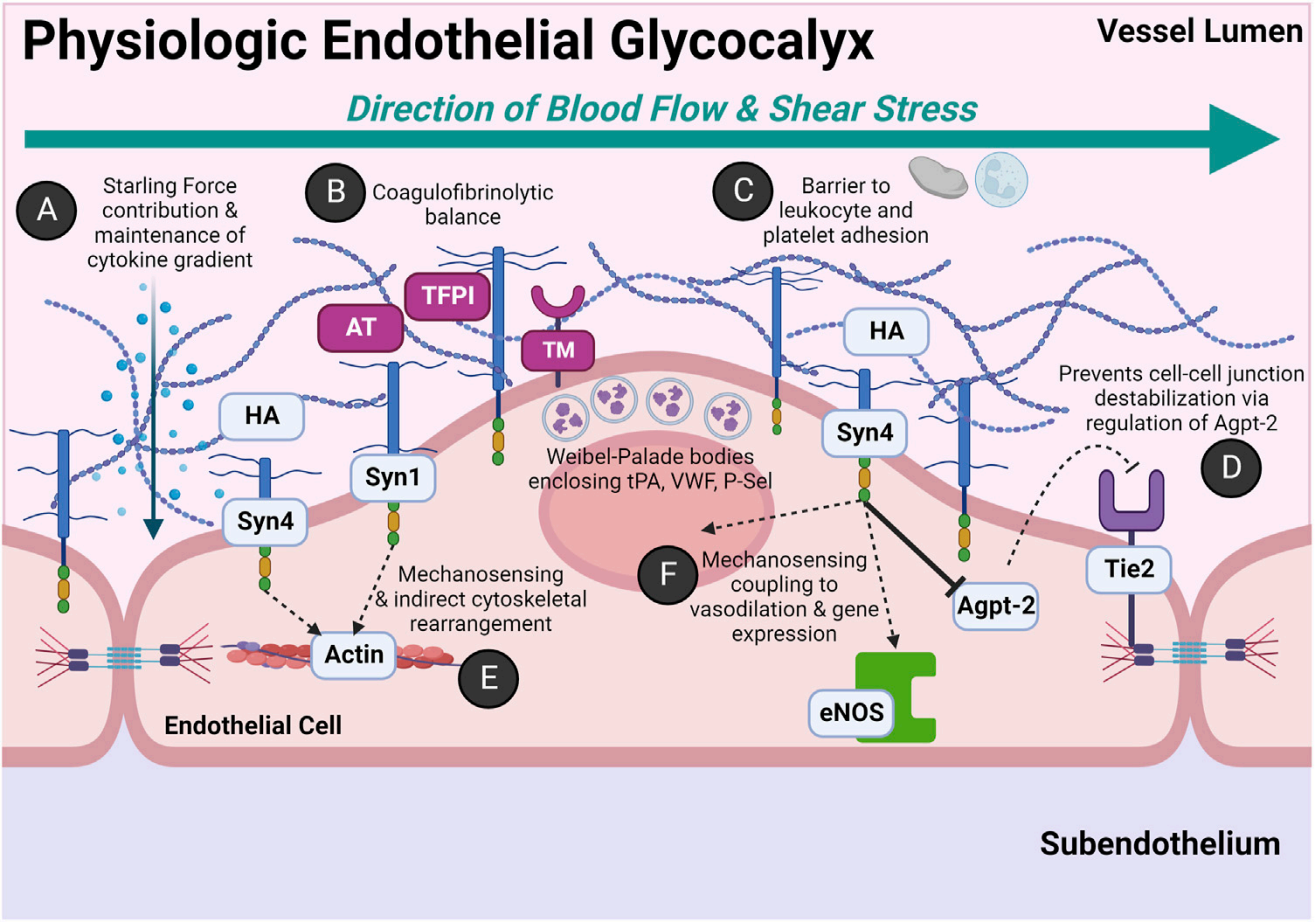
Physiologic Roles of the Endothelial Glycocalyx: An Anti-thrombogenic and Anti-adhesive Surface with Rapid Stress-sensing Capability. The glycocalyx is comprised of heparan sulfate proteoglycans (HSPGs) and glycosaminoglycans (GAGs). The HSPGs include the four transmembrane syndecans (Syn1-Syn4) and glypican. The primary GAGs include heparan sulfate (HS) and hyaluronan (HA). HS accounts for ∼50% of the GAG composition in the glycocalyx and covalently bonds to the Syn family and glypican. Other GAGs not pictured include keratan sulfate, dermatan sulfate, and chondroitin sulfate. **(A)** The negatively charged, hydrophilic moieties of HS and HA have variable cleavage lengths and post-translational modifications which confer significant degrees of specificity for binding cytokines and chemokines. This moderates oncotic and hydrostatic contributions of Starling Forces. Additionally, this creates a gradient of growth factors and signaling molecules which can be indirectly altered by constitutive or stress-induced transient changes in HSPG and GAG composition either by increased synthesis or enzymatic cleavage. **(B)** The endothelial glycocalyx maintains a gradient of endothelial-synthesized anticoagulant coagulofibrinolytic mediators. In addition to HS, antithrombin III (AT) and tissue factor pathway inhibitor (TFPI) are constitutively expressed in the endothelial glycocalyx. AT complexes with HS to inactivate many coagulation factors, primarily thrombin and Factor X. The anticoagulant TFPI complexes with and inactivates tissue factor (TF)-FVII complex and prothrombinase complex (FVa-FXa). The endothelium also constitutively expresses membrane-bound receptor thrombomodulin (TM), which in the presence of its ligand thrombin, activates circulating plasma protein C. Activated protein C inactivates circulating Factors V and VIII and also inhibits the anti-fibrinolytic, plasminogen activator inhibitor-1 (PAI-1). Weibel-Palade bodies (WPB) also play a significant role in coagulofibrinolytic balance, particularly for the activated endothelium (e.g., by endothelial agonists such as circulating plasma epinephrine). Seminal hemostatic mediators in WPB include tissue plasminogen activator (tPA), von Willebrand Factor (VWF), and P-selectin (P-Sel). **(C)** The glycocalyx serves as a physical barrier to leukocyte and platelet adhesion in the event adhesion molecule expression is induced on the endothelial luminal surface (e.g., P-selectin, ICAM-1, VCAM-1). **(D)** Syn4 *via* intracellular syntenin and synectin indirectly regulates angiopoietin-2 (Agpt-2) activity. High Agpt-2 levels antagonize the tyrosine kinase receptor Tie2, which subsequently destabilizes endothelial cell-cell junctions. Agpt-2 may also be found in WPBs. **(E)** Syn1 and Syn4 transcellularly signal luminal shear stress to rearrange the endothelial cytoskeleton. **(F)** Shear stress signaling by the intracellular domain of Syn4 also induces vasodilation *via* activation of endothelial nitric oxide synthase (eNOS). Shear stress also increases nuclear expression of genes such as those implicated in inflammation. For example, increased shear stress has shown to induce VCAM-1 and ICAM-1 expression. (Lindahl et al., 1998; Kolářová et al., 2014; Rayahin et al., 2015; Leligdowicz et al., 2018; Richter and Richter, 2019; Woodcock and Michel, 2021; Neubauer and Zieger, 2022). Abbreviations: Agpt-2, Angiopoietin-2; AT, Antithrombin III; eNOS, Endothelial Nitric Oxide Synthase; HA, Hyaluronan; Syn1, Syn4, Syndecan; TFPI, Tissue Factor Pathway Inhibitor; Tie2, tyrosine kinase receptor Tie2; TM, Thrombomodulin. Created with BioRender.com.

The endothelium is functionally and physically distinct and should thus be regarded as an independent organ system (Aird, 2004). Figure 2 depicts the physiology of the endothelium salient to this review. Table 1 further details biomarkers unique to the endothelial glycocalyx and endothelial-derived coagulofibrinolytic mediators.

**TABLE 1:** Endothelial Biomarkers and Coagulofibrinolytic Mediators and their Physiologic, Pathophysiologic, and Clinical Significance.

Biomarker/Coagulofibrinolytic mediator	Significance
Endothelial luminal layer	
Syndecans (Syn1- Syn4)	- Transmembrane structural heparan sulfate (HS) proteoglycans (HSPGs) that interact with surface receptors and transmit extracellular signals. Differing HS lengths and post-translational modifications alter plasma protein interactions and cytokine/chemokine gradients within the glycocalyx.
	- Syn1 and Syn4 provide shear mechanosensing and stimulate cytoskeletal remodeling
	- Loss of syndecan in the glycocalyx has demonstrated instability of the cytoskeleton and dysregulation of vasoactivity by decoupling shear stress from endothelial nitric oxide synthase (eNOS) activation.
	- Increased soluble Syn1 is a marker of severe systemic inflammation and is associated with illness severity.
	- Syn4 regulates angiopoietin-2 release from the endothelium which helps maintain endothelial cell-cell junctions Lindahl et al. (1998); Tkachenko et al. (2005); Reitsma et al. (2007); Yen et al. (2015); Farrugia et al. (2018); Richter and Richter, (2019)
Glypican	- Extracellular structural HSPG
	- Increased circulating levels in severe illness and inflammation.
	- Has been shown to increase eNOS activation in response to shear stress. Mellion et al. (1981); Zeng and Tarbell, (2014); Zeng and Liu, (2016); Richter and Richter, (2019)
Heparan Sulfate (HS)	- Glycosaminoglycan (GAG) which comprises ∼50% of the glycocalyx GAGs; highly variable in size
	- Covalently bonds to HSPGs glypican and Syn1-Syn4
	- Variable post-translational modifications alter plasma protein interaction with high specificity.
	- Negatively charged molecules contributing to oncotic and hydrostatic regulation of Starling forces.
	- Cleaved into varying lengths by heparanase-1 which serve as local cytokines to increase inflammation, endothelial permeability, and auto-heparinization.
	- Removal of HS from HSPGs by heparanase-1 increases glycocalyx degradation by matrix metalloproteinases (MMPs) and exposes P-selectin and cellular adhesion molecules for platelet and leukocyte adhesion/activation.
	Lindahl et al. (1998); Kolářová et al. (2014); Richter and Richter, (2019)
Hyaluronan (HA)	- High molecular weight (HMW) GAG which interacts with CD44 and other constituents of the glycocalyx.
	- Provides structural and lubricating effects.
	- Increased circulating levels in critical illness
	- Negatively charged molecules contributing to oncotic and hydrostatic regulation of Starling forces.
	- Cleaved by hyaluronidase or reactive oxygen species to low molecular weight (LMW) hyaluronan or HMW hyaluronan.
	- LMW hyaluronan has pro-inflammatory effects including activation of M1 phenotype in macrophages and acts as a ligand of endothelial receptors of TLR4, CD44, and receptor of hyaluronan-mediated motility (RHAMM).
	- HMW hyaluronan appears to have an anti-inflammatory effect by activating the M2 phenotype of macrophages. May also act as a mechanosensor to activate eNOS in response to shear stress. Kolářová et al. (2014); Nelson et al. (2014); Schmidt et al. (2014); Rayahin et al. (2015); Richter and Richter, (2019)
Chondroitin Sulfate	- GAG that when soluble may have anti-inflammatory effects *via* histone binding and NF-kB downregulation.
	- May have antibacterial properties *via* peptide inhibition Nelson et al. (2008); Kolářová et al. (2014); Richter and Richter, (2019)
Dermatan Sulfate	- GAG which may increase local cellular adhesion molecule expression and increase FGFR-dependent cell proliferation around epidermal injuries Penc et al. (1998); Penc et al. (1999); Richter and Richter, (2019)
Antithrombin III (AT)	- Liver- and endothelium-derived plasma anticoagulant protein which complexes with specific endogenous HS in the glycocalyx and blocks the active sites of thrombin and Factors IXa, Xa, XIa, and XIIa Ofosu et al. (1984); Anastasiou et al. (2012); Yen et al. (2015); Richter and Richter, (2019); Neubauer and Zieger, (2022)
Thrombomodulin (TM)	- Endothelial constitutively synthesized membrane-bound thrombin receptor which, when bound to thrombin, has a higher affinity for activation of the anticoagulant protein C
	- Has a binding site separate from that of protein C for activation of thrombin-activatable fibrinolysis inhibitor (TAFI). The dual and counteracting pathways between protein C activation and TAFI dictated by thrombomodulin are termed the “thrombomodulin-thrombin switch” which is also influenced by a endothelial-derived cofactor named the endothelial protein C receptor (EPCR). The thrombomodulin-thrombin switch is likely a misnomer implying a competitive either-or state of the “switch”, when in reality aPC and TAFI are likely in a dynamic equilibrium influenced by transient changes in thrombin, EPCR and other cofactor concentrations and post-translational modifications
	- Increased plasma levels (termed soluble thrombomodulin [sTM]) are measurable in shock states as a marker of endotheliopathy; increased sTM levels prognosticate illness severity Yen et al. (2015); Richter and Richter, (2019); Hatton et al. (2021); Kregel et al. (2022)
Tissue Factor Pathway Inhibitor (TFPI)	- Endothelial constitutively synthesized anticoagulant protein which binds and blocks coagulation activity of Tissue Factor (TF)-FVIIa complex and prothrombinase complex (FVa-FXa)
	- Interacts with HS of the endothelial glycocalyx
	- Found intracellularly in quiescent platelets; high levels are also found in placenta and myometrium Girard and Broze, (1993); Kuczyński et al. (2002); Xiong et al. (2010); Yen et al. (2015); Mast, (2016); Richter and Richter, (2019); Neubauer and Zieger, (2022)
Ectonucleoside triphosphate diphosphhydrolase-1 (E-NTPDase1/CD39)	- Constitutively expressed membrane-bound anti-thrombotic enzyme which converts adenosine diphosphate (ADP) and adenosine triphosphate (ATP) into adenosine. ADP is a potent platelet activator released from platelet alpha granules. ATP, ADP, and adenosine monophosphate (AMP) are also released from resting endothelial cells at low rates, but increased release occurs with stress states. Marcus et al. (1991); Deaglio and Robson, (2011); Neubauer and Zieger, (2022)
Weibel-Palade Bodies (WPB)	
von Willebrand Factor (VWF)	- WPBs fuse with the endothelial cell membrane to undergo exocytosis of VWF in response to increased or decreased shear stress, hypoxemia, or vasoactive signaling molecules such as catecholamines, bradykinin, histamine, vasopressin, and thrombin
	- VWF facilitates platelet adhesion by serving as a bridge between subendothelial collagen and platelet receptor GPIbα Pinsky et al. (1996); McCormack et al. (2017); Neubauer and Zieger, (2022)
Tissue Plasminogen Activator (tPA)	- Serine protease which is constitutively secreted by endothelial cells, but also transiently increased by exocytosis of stores in WPBs; high concentrations are particularly found in cells of precapillary arterioles
	- Organs which include a high tPA content include the heart, lung, kidney, and brain
	- Pro-fibrinolytic which converts liver-derived circulating plasminogen into plasmin. Plasmin then serves multiple functions: cleavage of fibrin (ogen), degradation of extracellular matrix by activation of metalloproteinases, and activation of growth factors
	- Inactivated by PAI-1, PAI-2
	- Plasma half-life of approximately 3 min and averages a concentration of 5 ng/ml; cleared primarily by the liver
	- Enzymatically active tPA also packaged in pre-synaptic vesicles and secreted by perivascular sympathetic nerve fibers directly into the microvasculature and small arteriole walls
	- At the blood-brain barrier, tPA is a direct modulator of neurovascular coupling by acting as an agonist at the luminal endothelial N-Methyl-D-Aspartate (NMDA) receptor to increase NO synthesis with subsequent increase in cerebral blood flow.
	Huber et al. (2002); O'Rourke et al. (2005); Su et al. (2009); Haile et al. (2012); Kwaan (2014); Yepes, (2015); Fritsma (2020); Moore (2022); Neubauer and Zieger, (2022)
P-Selectin (P-Sel)/CD62P	- Enables leukocyte adhesion to the vessel wall *via* interaction with P-selection glycoprotein ligand-1 (PSGL-1) on the leukocyte surface
	- Induces TF expression on monocytes
	- Also found in platelet α-granules and expressed by activated platelets to mediate leukocyte-platelet binding
	- Increased circulating plasma levels are detectable in thrombotic and/or endotheliopathic conditions André, (2004)
Angiopoietin-2 (Agpt-2)	- Endothelial constitutively synthesized and secreted autocrine signaling molecule on the tyrosine kinase receptor Tie2
	- At resting state, Agpt-2 remains at low concentrations and demonstrates agonistic functions at heterodimeric Tie1-Tie2 receptors in concert with Agpt-1 agonism. Tie2 agonism reinforces endothelial survival and endothelial cell-cell junctions
	- Endothelial cell activation sheds Tie1 from Tie2, and together with exocytosis of WPB and higher concentration of Agpt-2, Agpt-2 switches to an antagonist of Tie2 and destabilizes endothelial cell-cell junctions
	- Syn4 indirectly regulates Agpt-2 release *via* intracellular association with syntenin and synectin, intracellular molecules important for WPB biosynthesis
	- In sepsis, increased Agpt-2 level is associated with increased mortality, tissue hypoperfusion, organ dysfunction, coagulopathy, and inflammation
	- Plays a pivotal role in capillary permeability and leak in condition such as sepsis and acute respiratory distress syndrome Ju et al. (2014); Leligdowicz et al. (2018); Richter et al. (2022b)
Endothelial Intracellular	
Endothelial Nitric Oxide Synthase (eNOS)	- Generates nitric oxide (NO) in response to shear stress to cause vasodilation. eNOS can also be activated by serotonin, VEGF, bradykinin, and adenosine.
	- NO serves as an inhibitor of WPB exocytosis.
	- NO diffuses into the bloodstream to prevent platelet activation indirectly *via* increasing platelet intracellular cGMP which then inhibits release of stored intracellular Ca^2+^ Radomski et al. (1987); Radomski et al. (1990); Matsushita et al. (2003); Neubauer and Zieger, (2022).
Plasminogen Activator Inhibitor-1 (PAI-1)	- Also referred to historically as endothelial cell-type plasminogen activator inhibitor
	- Anti-fibrinolytic that inhibits tPA and uPA by forming complexes which are then cleared by the liver
	- Classified as an acute phase reactant and synthesis is stimulated by inflammatory cytokines and growth factors such as IL-1, TNFα, TGFβ, estrogen, thrombin, insulin, and angiotensin II
	- Synthesized transiently by endothelial cells, contained in α-granules of quiescent platelets, and synthesized by hepatocytes Sprengers and Kluft, (1987); Vaughan, (2005); Neubauer and Zieger, (2022)
Intercellular Adhesion Molecule-1 (ICAM-1)/Vascular Cellular Adhesion Molecule-1 (VCAM-1)	- Synthesis and expression of endothelial cell surface glycoproteins ICAM-1 and VCAM-1 are inducible by TNFα and other inflammatory cytokines, reactive oxygen species, hyperglycemia, toll-like receptor agonists, and shear stress
	- Enable leukocyte adhesion to vessel wall Kong et al. (2018)

How this large, complex multifarious system communicates nearly immediately across its vast length and surface area is an impressive capability, the understanding of which is still growing, and which finds importance in the speed with which a patient with SHINE can either deteriorate or respond to early resuscitation. The collective endothelial surface of up to 7,500 m^2^ transmits messages from one part of the body to another *via* many pathways (Dobson et al., 2015). The first and most obvious pathway is the transmission of a drop in pressure from one vascular bed to another which is nearly immediate in a closed pressure system. Additionally, it has been demonstrated that direct electrical stimulation of the microvasculature causes local vasoconstriction; however, the endothelium and subendothelial smooth muscle cells propagate the depolarization along the vessel axis to cause long distance vasodilation primarily mediated by voltage-gated calcium channels and increased nitric oxide generation induced by the calcium influx (Figueroa et al., 2007).

The nearly immediate long distance signaling by the endothelium has also been observed for fibrinolysis. Hau Kwaan first noted in the 1950s that stimulation of one venous segment elicited fibrinolytic activities from another vein located far from the site of stimulation (Kwaan, 2014). This implied that there was a possible transmission of hemostatic and inflammatory markers to the intravascular space which can alter endothelial physiology at sites distant from focal injury. However, it later became apparent that perivascular sympathetic pathway activity was another pathway responsible for near-immediate signal transmission (O'Rourke et al., 2005; Kwaan, 2014). Therefore, the transmission of information regarding inflammation and thrombosis in SHINE is a function of not only the standard macrovascular and microvascular pressure gradients, and concomitant shear and blood chemistry, but also of the microvascular Starling forces and sympathetic electrical activity which in concert achieve a delicate immuno-thrombotic balance designed to simultaneously preserve microvascular flow and tissue perfusion during the development of SHINE.

Even though the concept of SHINE has only recently been proposed, earlier literature eloquently defined the endothelium’s importance. In 1982, Alfred Fishman wrote: “In recent years, as the tempo of fresh insights into the complexity of the endothelium has increased, realization has dawned that instead of serving simply as an inert barrier between blood and tissues, the endothelium is a distributed organ of considerable biological potential that not only extends throughout the body in the convenient form of an anti-thrombogenic vascular lining but also performs other distinctive biologic functions at different vascular sites and individual organs” (Fishman, 1982). For example, the endothelium of the pulmonary capillaries has evolved to enhance gas and water exchange while the endothelium of the aorta has evolved to withstand the high pressures exerted by the pumping force of the heart. This “organ” of large surface area has important physiologic functions such as maintaining blood viscosity, providing nutrients, modulating vasomotor function, and participating in immunological surveillance by maintaining innate and acquired immunity while orchestrating a communication between tissue perfusion and tissue flow (Fishman, 1982; Aird, 2006; Johansson et al., 2017a).

## 2 Pathophysiology of SHINE

Determinants of blood flow include the vessel cross-sectional area, perfusion pressure, and blood viscosity (Rosato et al., 1968). Adaptive responses to decreased perfusion often become maladaptive in the shock state. For example, patients in septic shock experience systemic vasodilation which worsens tissue perfusion and warrants treatment with fluids and vasopressors. Similarly, hypocoagulability and hyperfibrinolysis in patients with shock may represent a maladaptive attempt to restore perfusion by decreasing blood viscosity against a pro-thrombotic endothelium.

**FIGURE 3:**
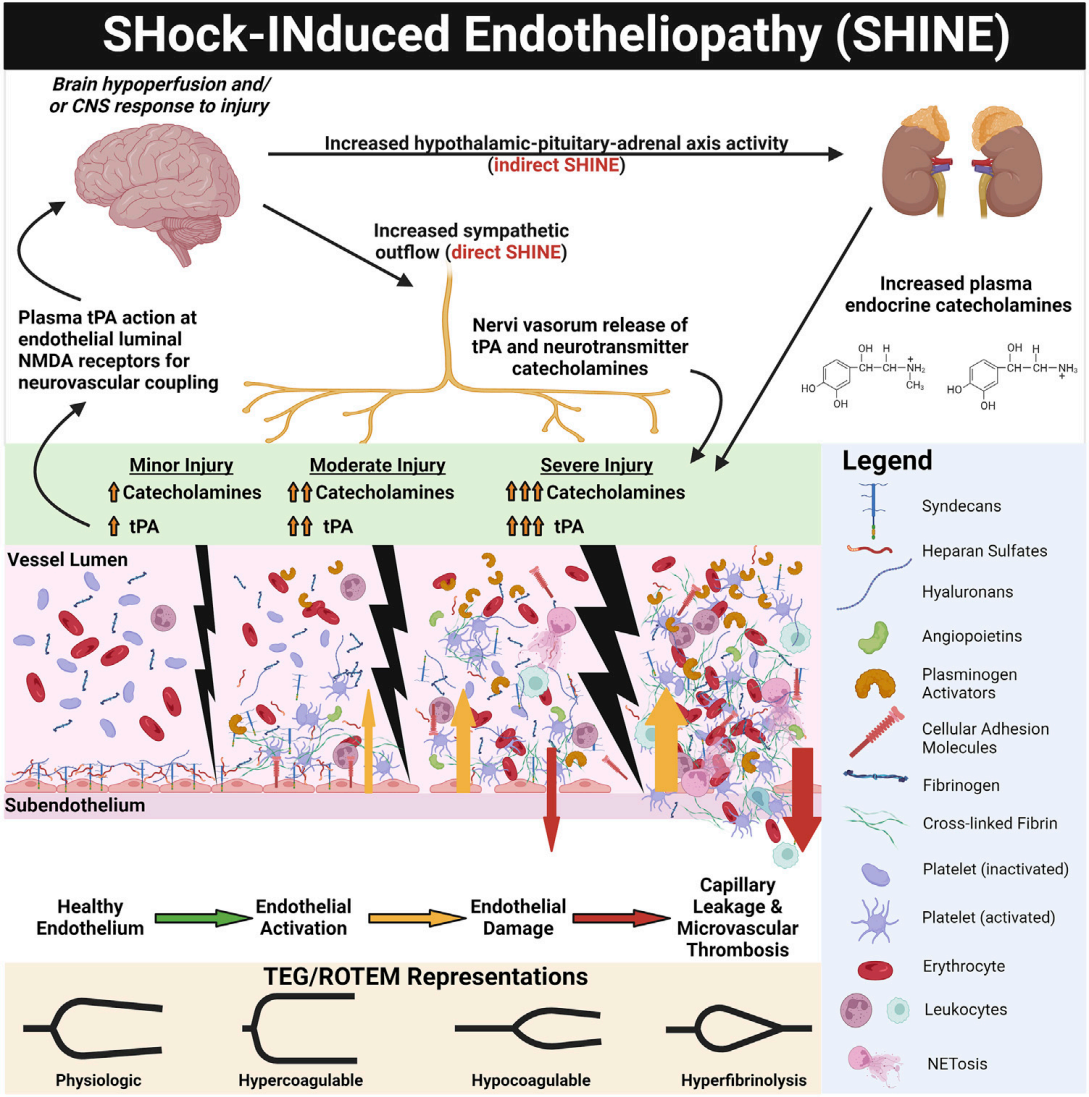
SHock-INduced Endotheliopathy (SHINE) as a Reflection of Injury Severity. Increasing sympatho-adrenal activation with increasing injury and shock severity leads to endothelial activation and damage. Increased sympathetic outflow directly provokes SHINE *via* perivascular sympathetic nerve exocytosis of neurotransmitter catecholamines and enzymatically active tissue plasminogen activator (tPA) into the vessel walls and directly into the microvasculature (O'Rourke et al., 2005; Kwaan, 2014). Hypothalamic-pituitary-adrenal axis activity also increases circulating plasma catecholamines. The corresponding endothelial and hemostatic changes are dose-dependent to injury/shock severity, as measured by endothelial biomarkers (e.g., plasma syndecan-1 and soluble thrombomodulin) and on thromboelastography (TEG) and rotational thromboelastometry (ROTEM) tracings. For example, with trauma, TEG/ROTEM tracings progress from physiologic hemostasis to hypercoagulable in mild trauma, to hypocoagulable in moderate trauma, and finally hyperfibrinolytic in severe trauma (Johansson and Ostrowski, 2010). Genetically preserved responses to critically ill patients inflicted by trauma, burns, and sepsis are similar, suggesting early responses to shock are evolutionarily preserved wherein SHINE may be a unifying mechanism (Xiao et al., 2011; Johansson et al., 2017a). The catecholaminergic surge (in particular the vasoconstrictive action of norepinephrine) causes glycocalyx shedding, endothelial injury, and de-endothelialization of perfused vessels (Dolgov et al., 1984; Makhmudov et al., 1985; Kristová et al., 1993; Vischer and Wollheim, 1997). The activated/injured endothelium promotes thrombosis, causing occlusion of the microvasculature. Together with capillary leak, perivascular edema, and vasoconstriction, these vascular responses provoke a cycle of progressive tissue hypoperfusion, hypovolemia, organ injury, and increasing sympatho-adrenal activation (Opal and Van Der Poll, 2015; Johansson et al., 2017a). It has been hypothesized that the ensuing hypocoagulability and hyperfibrinolysis may be a compensatory counterbalance to the pro-thrombotic endothelium in an attempt to maintain patency of the microvasculature (Johansson and Ostrowski, 2010). Therefore, the two major hemostatic compartments—the endothelium and the blood—may “switch” phenotypes in some progressing shock states. Whereby the physiologic endothelium acts as anti-thrombogenic surface to oppose coagulable blood, in shock, the roles may switch to a pro-thrombotic endothelium with a hypocoagulable/hyperfibrinolytic blood phenotype in attempt to rebalance hemostasis, decrease the blood viscosity, and restore perfusion (Johansson and Ostrowski, 2010) (see Figure 4). Not only does tPA exert pro-fibrinolytic activity *via* enzymatic activation of plasminogen, but tPA in the brain uniquely acts as a signaling agonist on the N-Methyl-D-Aspartate (NMDA) receptor on the endothelial luminal surface of small cerebrovascular arterioles. The activated NMDA receptor increases synthesis of nitric oxide to cause vasodilation and increase cerebral blood flow (Su et al., 2009; Haile et al., 2012; Yepes, 2015). Thus, increased free tPA (that is, free from complexes with PAI-1 and other inhibitors) in shock states may simultaneously increase systemic perfusion *via* fibrinolysis of occlusive thrombi and as a neurovascular coupling agent to increase cerebral blood flow (Su et al., 2009; Yepes, 2015). Created with BioRender.com.

Critically ill patients in shock demonstrate endothelial injury and hypocoagulability/hyperfibrinolysis directly proportional to the severity of disease and can be predicted by injury severity score (ISS) or base deficit (BD) in trauma (Johansson et al., 2010; Johansson and Ostrowski, 2010; Moore et al., 2016b; Johansson et al., 2017c), the Sequential Organ Failure Assessment (SOFA) score in septic patients (Ostrowski et al., 2015; Mochizuki et al., 2018; Iba et al., 2019a; Berthelsen et al., 2019; Bestle et al., 2020), and time until return of spontaneous circulation (ROSC) in patients who suffer from PCAS (White et al., 2011; Wada, 2017). Sympatho-adrenal activation (as measured by increased plasma catecholamines) likewise associates with the degree of endothelial injury, increasing injury/disease severity, and mortality risk (Dolgov et al., 1984; Makhmudov et al., 1985; Johansson et al., 2012; Johansson et al., 2015; Jung et al., 2015; Ostrowski et al., 2015; Di Battista et al., 2016; Johansson et al., 2017b). It has been posited that catecholamines, particularly the vasoconstrictive effect of norepinephrine, enact dose-dependent damage upon the endothelium to cause SHINE (Dolgov et al., 1984; Makhmudov et al., 1985; Kristová et al., 1993; Vischer and Wollheim, 1997; Johansson and Ostrowski, 2010). Beyond circulating endocrine catecholamines, peripheral sympathetic nerves may directly activate the endothelium and additional layers of blood vessels *via* neurotransmitter catecholamines (Peng et al., 2000). Perivascular sympathetic nerves can also release proteins such as tPA into small vessel walls and directly into microcirculation, serving as a backup of the endothelium to balance hemostasis (O'Rourke et al., 2005; Kwaan, 2014). tPA release may also be related to hypoperfusion (and sensing of decreased shear) independent of hypoxemia. Differentiating the effects of circulating catecholamines *versus* direct microvascular innervation in driving SHINE have not been previously evaluated and warrants future investigation. Additionally, whether increased catecholamines and SHINE are correlational markers of organ injury or causal pathophysiologic drivers remains the subject of further study. Figure 3 represents the prevailing pathophysiologic mechanism of SHINE.

**FIGURE 4:**
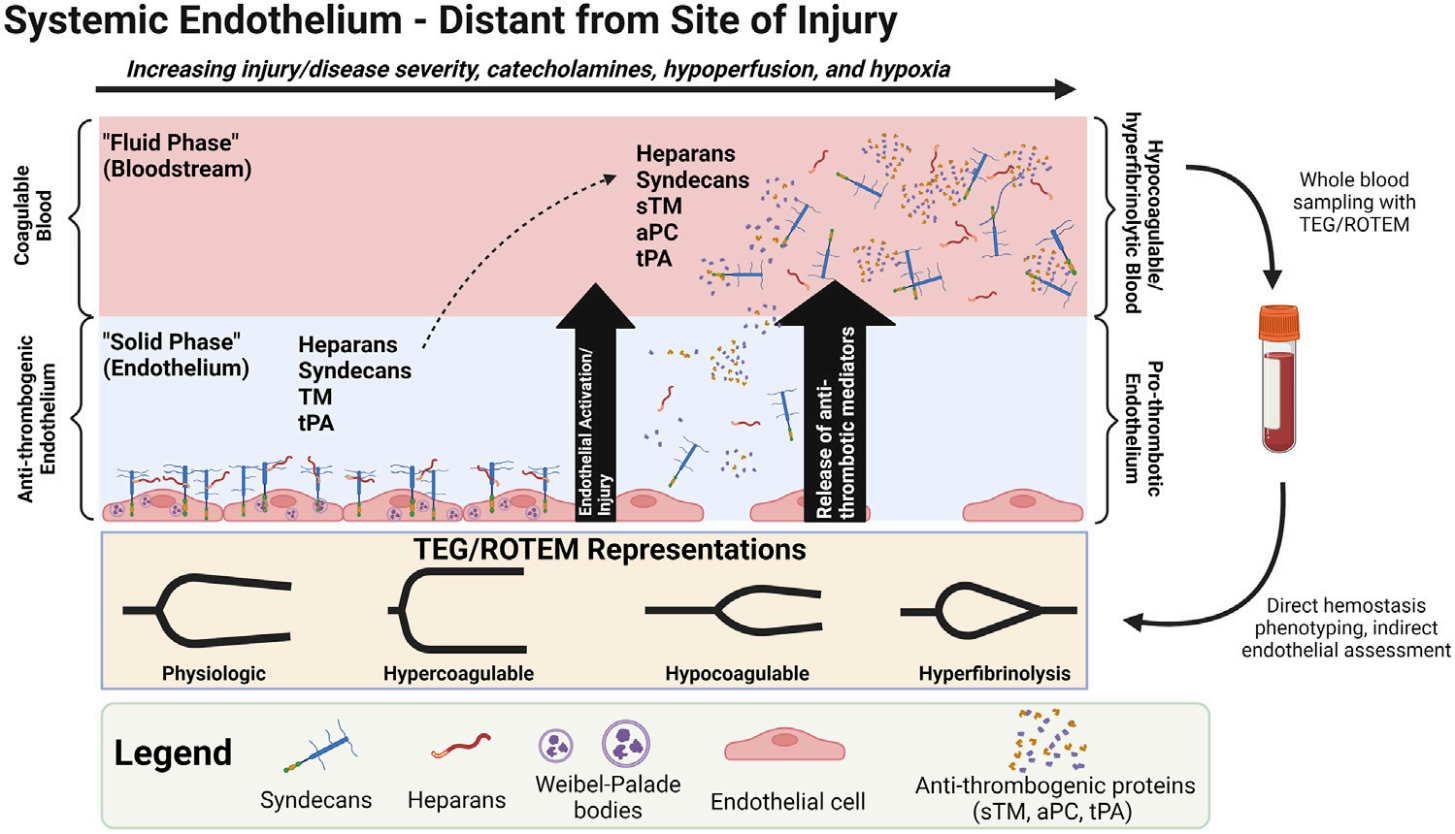
Shock-INduced Endotheliopathy (SHINE) “Phenotype Switching” *via* Release of Anti-thrombogenic Mediators from the Endothelium to the Bloodstream. One possible contributor of the hypocoagulable/hyperfibrinolytic phenotype in progressive shock may be the release of physiologically endothelial-sequestered anti-thrombogenic mediators to the bloodstream during SHINE when the endothelium is systemically activated and/or injured. Note that protein C is physiologically a plasma protein, but increases in soluble thrombomodulin (sTM) may increase the conversion of protein C to activated protein C (aPC). (Johansson and Ostrowski, 2010). Abbreviations: aPC, activated Protein C; ROTEM, Rotational Thromboelastometry; sTM, soluble Thrombomodulin; TEG, Thromboelastography; tPA, tissue Plasminogen Activator. Created with BioRender.com.

Hypocoagulability/hyperfibrinolysis in shock states also correlates to biomarkers of endothelial glycocalyx derangement, such as increased circulating Syn-1, soluble TM (sTM), P-selectin, and an increased ratio of Agpt-2:Agpt-1 (Ostrowski et al., 2015; Johansson et al., 2017a; Johansson et al., 2017b). Glycocalyx disruption and endotheliopathy contributes to a cycle of increasing tissue hypoxia, capillary leak, micro-thrombosis, organ failure, and mortality in patients with shock (Faust et al., 2001; Adrie et al., 2002; Adrie et al., 2004; Neumar et al., 2008; Johansson and Ostrowski, 2010; Gando et al., 2011; Holcomb, 2011; Cohen et al., 2012; Levi et al., 2012; Angus and Van der Poll, 2013; Kim et al., 2013; Cohen et al., 2015; Opal and Van Der Poll, 2015; Johansson et al., 2017a). Thus, the hypocoagulable and hyperfibrinolytic state in progressive shock may be a counterbalance to the pro-thrombotic endothelium in an attempt to restore perfusion to vital organs (Johansson and Ostrowski, 2010). This counterbalance may be viewed as an attempt to rebalance hemostasis by switching phenotypes of the endothelium and blood (Johansson and Ostrowski, 2010). In homeostasis, the endothelium is an anti-thrombogenic and anti-adhesive surface to balance with the coagulable blood. These phenotypes may “switch” in SHINE whereby the hypocoagulable/hyperfibrinolytic blood may counteract the pro-thrombotic endotheliopathy. This may be rationalized as the physiologically sequestered anti-thrombogenic players (e.g., endogenous heparans, TM, tPA) being released from the activated/injured endothelium into the bloodstream (Figure 4). In turn, sTM promotes hypocoagulability *via* increased activation of protein C to activated protein C (aPC) which then inactivates Factor V (FV) and Factor VIII (FVIII). APC also inhibits plasminogen activator inhibitor-1 (PAI-1). The increase in tPA may also overwhelm circulating PAI-1 levels to provoke a pro-fibrinolytic phenotype. The glycocalyx layer may also become hyperpermeable because of sialidase-mediated disruption of the endothelial border in shock. Although not particularly evaluated for their role in shock, it has been reported that sialic acid residues embedded in the glycocalyx layer regulate the permeability of microvascular structures (Betteridge et al., 2017).

Moreover, tPA uniquely in the cerebrovasculature may counteract the vasconstrictive effects of norepinephrine to restore brain perfusion *via* tPA’s agonist action upon the N-Methyl-D-Aspartate (NMDA) receptor on the endothelial luminal surface of the blood-brain barrier (BBB), causing NO synthesis and vasodilation of cerebral arterioles (Su et al., 2009; Yepes, 2015). Thus, sympatho-adrenal-driven increases in tPA elicits at least two mechanisms to increase perfusion in shock states: 1) increased fibrinolysis to decrease blood viscosity and lyse occlusive thrombi, and 2) increased cerebral blood flow *via* direct cerebral arteriolar vasodilation.

Recently, increased attention to SHINE has elevated the perspective of the endothelium not merely as an anatomic entity, but as a unique organ with unique functions and biomarkers of injury which requires restoration in severe injury and disease (Aird, 2006; Dobson et al., 2015; Johansson et al., 2017a). The systems hypothesis of trauma (SHOT) has questioned the increasingly reductionist approach to trauma resuscitation, highlighting that hemorrhaging trauma patients are still expiring perhaps due to overemphasis on symptomatic care rather than addressing the underpinning system derangements associated with severe injury (Dobson et al., 2022). SHOT posits the endothelium as the “systems integrator” critical for veno-arterial coupling and preserving the blood-brain barrier essential for maintaining the brain’s privilege over the entire body. Thus, the endothelium may be one system which necessitates resuscitation to switch from the injury phenotype to that of survival (Dobson et al., 2022).

The depth and duration of shock may be evaluated with endothelial biomarker assessment and adjunctive VHAs to guide early restorative therapies. For example, in SHINE associated with TIC, prehospital transfusion of plasma demonstrates a protective effect on the injured endothelium with salutary restitution of the glycocalyx layer for patients who required massive transfusion (MT) (Moore et al., 2018; Sperry et al., 2018; Pusateri et al., 2020). Similar salutary benefit has shown to prevent endothelial damage in early sepsis when these patients are administered therapeutic plasma exchange (TPE) with fresh frozen plasma (Drost et al., 2021; Pape et al., 2021; Stahl et al., 2021). Heparanase-1 is a primary mediator for endothelial injury particularly early in sepsis, whereas heparanase-2 is a protective mediator. Recent studies have shown that shedding of the endothelial glycocalyx, as well as the ratio of heparanase-1 to heparanase-2, is diminished in septic patients who receive early TPE within the first 6 h of presentation. Therefore, it has been proposed that direct endothelial glycocalyx assessment and surrogate assays for shredded endothelial glycocalyx, as well as assays for heparanase-1 and heparanase-2 levels, enable early detection of SHINE to guide earlier antibiotics and targeted endothelial therapy with TPE (Drost et al., 2021; Pape et al., 2021; Stahl et al., 2021). VHAs assist the early detection of coagulopathies associated with SHINE when these patients are treated with plasma whether for trauma or sepsis (Thölking et al., 2015; Moore et al., 2018; Sperry et al., 2018; Pusateri et al., 2020). Recent trauma models adopting VHAs show promise for earlier identification of hemostatic derangement and SHINE, which predicts the need for MT in trauma patients (Thorn and Maegele, 2021). Likewise, early use of VHAs in SIC enables early detection of the depth and duration of shock prior to coagulopathic manifestation by conventional coagulation tests (CCTs) and other standard clinical and biologic markers (Pavoni et al., 2020). Combined with CCTs, VHAs, and clinical and laboratory markers such as lactate and procalcitonin, a PBM approach to assess the depth and duration of shock is becoming a reality whether in TIC or SIC (Vorweg et al., 2013; Müller et al., 2014; Schöchl and Schlimp, 2014; Saraiva et al., 2016; Johansson et al., 2017a; Martínez et al., 2018; Stettler et al., 2019; Walsh et al., 2020b; Scarlatescu et al., 2020; Carge et al., 2021; Drost et al., 2021; Pape et al., 2021; Tuan et al., 2021; Bunch et al., 2022a).

The direction of coagulation is driven not only by the coagulofibrinolytic mediators, but the endothelium is also influenced by the nature and severity of the initial injury, timing and methods of resuscitation, genetic hematologic makeup of the patient, underlying conditions (e.g., age, gender, atherosclerotic risk factors) and medications, all of which determine whether the patient’s endothelium and blood will respond with either a pro- or anti-thrombotic phenotype (Mellion et al., 1981; Kaplanski et al., 1998; Levi and van der Poll, 2008; Park et al., 2014; Dobson et al., 2015; Moore et al., 2016b; Strandin et al., 2016; Moore et al., 2019b; Wernly et al., 2019; Richter et al., 2022a). For example, in hemorrhagic shock, crystalloid resuscitation has demonstrated deleterious effects on the endothelium whereas plasma resuscitation has shown to restore it (Moore et al., 2018; Sperry et al., 2018; Brill et al., 2021). VHAs such as TEG and ROTEM provide a macroscopic, actionable indication of blood hemostatic integrity and severity of endothelial injury (Saini et al., 2019; Kim et al., 2021). Real-time VHA monitoring of coagulopathies associated with SHINE requires an understanding of the history, rationale, and efficacy of these tests.

### 2.1 VHAs as precision-based medicine tools to treat the spectrum of coagulopathic phenotypes associated with SHINE

Differing shock etiologies cause different early responses by the endothelium. These responses often share hemostatic phenotypes but also demonstrate unique aspects which implicate the need for personalized resuscitation of the endothelium. For example, the hypotensive septic shock patient often manifests acute hypofibrinolysis, or so-called “fibrinolytic shutdown”, and thus does not benefit from administration of the anti-fibrinolytic tranexamic acid (TXA). On the contrary, a patient in severe traumatic hemorrhagic shock with a hyperfibrinolytic phenotype may benefit from TXA (Moore et al., 2020). The hypotensive hypercoagulopathic patient in septic shock and the hypotensive hypocoagulopathic patient in hemorrhage-induced shock represent two opposite extremes along the coagulofibrinolytic spectrum of shock-associated coagulopathies.

SHINE can alter hemostasis on a minute-to-minute basis by endothelial reactions (e.g., shedding of the glycocalyx layer, or “phenotype switching” among pro- or anti-thrombotic and pro- or anti-fibrinolytic), causing worsening microvascular injury and organ malperfusion. Serial bedside VHAs offer more timely goal-directed blood component therapy (BCT) and hemostatic adjunct therapy (HAT) when these coagulopathies may switch phenotypes rapidly. Therefore, assessment of endothelial function and injury pertaining to hemostasis cannot solely rely upon the plasma-based CCTs prothrombin time (PT), activated partial thromboplastin time (aPTT), as well as platelet count, fibrinogen, and D-dimer. Rather, point-of-care global hemostasis assessment with whole blood VHAs, bedside assessment of organ perfusion, and other laboratory markers for hypoperfusion such as serial arterial base deficit and lactate, enable timely physiologic and targeted hemostatic resuscitation of patients in shock (Vorweg et al., 2013; Schöchl et al., 2014; Johansson et al., 2017a; Martínez et al., 2018; Walsh et al., 2020b; Bunch et al., 2022a).

Much as SHINE is a recently proposed framework for classifying patients with shock-associated coagulopathies, a simultaneous expansion has occurred for the use of VHAs to better define the phenotype of these coagulopathies and offer goal-directed therapy (Vorweg et al., 2013; Johansson et al., 2017a; Bugaev et al., 2020; Walsh et al., 2020b). VHAs required decades of guiding resuscitation in liver transplantation, cardiac surgery, and trauma before randomized controlled trials (RCTs) demonstrated the advantage of VHAs over CCTs alone for patients with hemorrhagic shock in these settings (Curry et al., 2018; Bugaev et al., 2020; Walsh et al., 2020b). To date, there remains no robust RCTs demonstrating superiority of VHAs to guide hemostasis management of patients in shock while on extracorporeal membrane oxygenation (ECMO) or with Left Ventricular Assist Devices (LVADs). However, VHAs are overwhelmingly used in these clinical settings (Colman et al., 2019; Bunch et al., 2021; Volod et al., 2022; Volod and Wegner, 2022). The clinician should not be dissuaded from using VHAs because of the absence of large RCTs demonstrating VHA utility to treat the hemostatic derangements caused by SHINE for etiologies other than liver transplantation, cardiac surgery, and trauma. In many other settings, VHAs have demonstrated utility by numerous observational and prospective studies (Adamzik et al., 2011; Brenner et al., 2012; Collins et al., 2014; Tran et al., 2015; Hans and Besser, 2016). The “one-size-fits-all” approach of large RCTs may hinder detection of the “signal from the noise” for the benefits of VHA-guided resuscitation for shock, especially in the care of complex patients because of infrequently met inclusion criteria. On the other hand, PBM allows for personalized treatment based on the patient’s individual phenotype. TEG/ROTEM enable both real-time identification of dynamic hemostatic phenotypes (phenotype switching) and provision of real-time guidance for the treatment of coagulopathies (individualized goal-directed resuscitation). (Maslove et al., 2017; Görlinger et al., 2019; McKinley et al., 2019; Stettler et al., 2019; Walsh et al., 2020b). Hence, VHAs may aid diagnosis and guide treatment for patients with all forms of SHINE. The adherence to the “one-size-fits-all” mandate by which large RCTs must establish clear statistical evidence prior to using a diagnostic test is challenged by the long history and evolution of VHAs as PBM tools in liver transplantation, cardiac surgery, trauma, and most recently, postpartum hemorrhage (PPH), ECMO, and LVAD resuscitation (Collins and Varmus, 2015; Beckmann and Lew, 2016; Letson and Dobson, 2017; Tignanelli and Napolitano, 2018; Stettler et al., 2019; Walsh et al., 2019; Bell et al., 2022).

**FIGURE 5:**
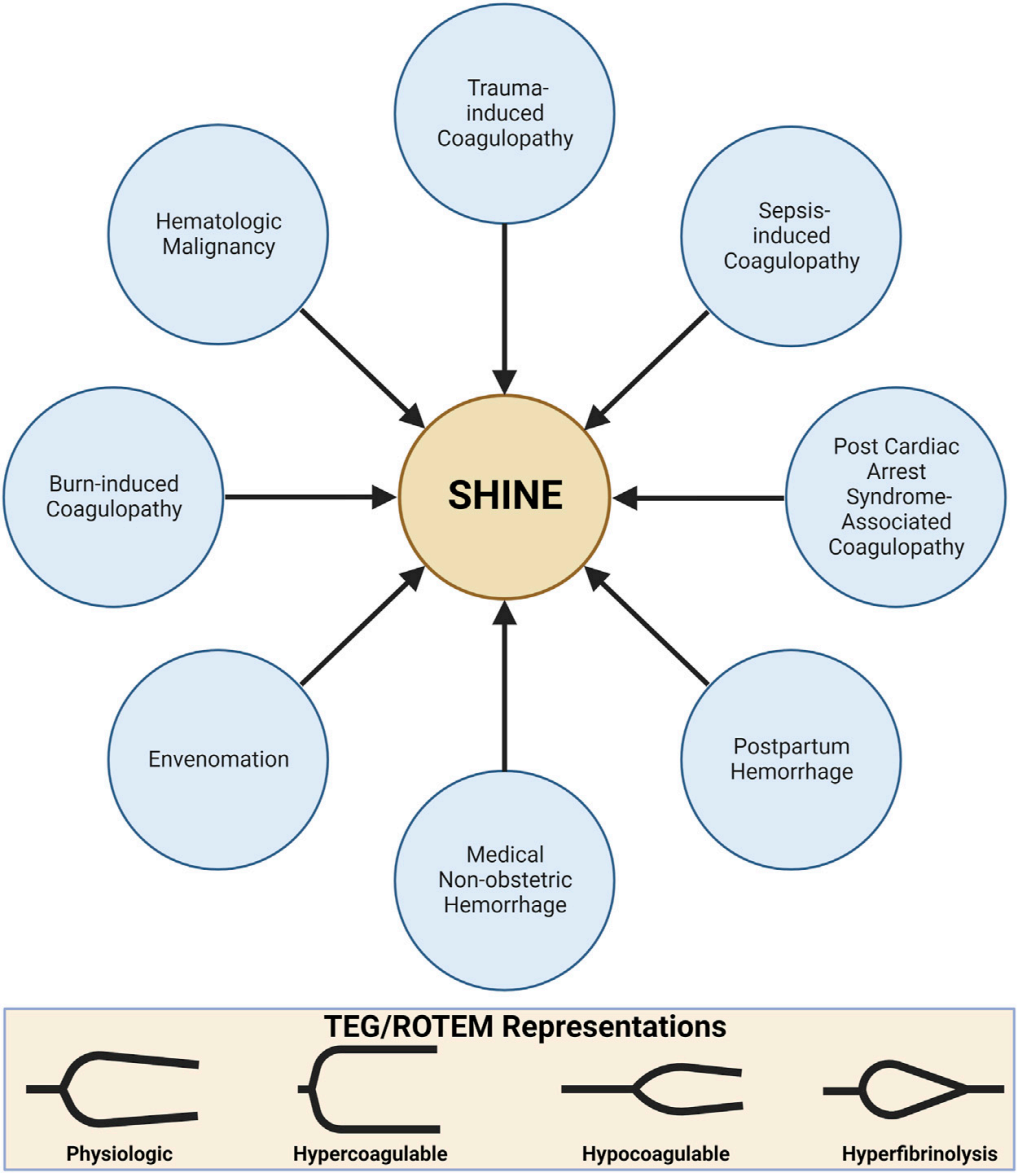
SHock-INduced Endotheliopathy (SHINE) as a Unifying Mechanism for Coagulopathies Associated with Critical Illness. In this review, we contextualize SHINE as defined by the viscoelastic hemostatic assays thromboelastography (TEG) and rotational thromboelastometry (ROTEM) within many causes of shock. Created with BioRender.com.

The remaining review describes the principles of VHAs and applying these principles to the monitoring and treatment of patients in shock afflicted by SHINE. Hemostatic phenotypes associated with SHINE are first delineated by applying the accepted pathophysiologic drivers of endotheliopathy for trauma-induced coagulopathy (TIC) and sepsis-induced coagulopathy (SIC). These prevalent causes of coagulopathy in critical illness set the foundation by which to contextualize the coagulofibrinolytic spectrum of SHINE. In turn, one may rationalize the application of VHAs for the diagnosis and treatment of all shock-associated coagulopathies. Here, we additionally emphasize VHAs for SHINE in post-cardiac arrest syndrome (PCAS), medical causes of hemorrhage, PPH, burns, and venom-induced consumption coagulopathy (VICC) (Vincent and De Backer, 2013; Standl et al., 2018). For many causes of SHINE, whether medical or surgical, the use of VHAs is in its relative infancy. The use of VHAs for these settings may be compared to the early days of liver transplantation which required years of study before large RCTs demonstrated overwhelming benefit (Kang et al., 1985; Starzl, 2002; Walsh et al., 2020b). This physiologic primer likewise serves as the cornerstone for future expansion of trials to determine the benefit of VHAs for management of patients with shock-associated coagulopathies regardless of the etiology. Briefly, we next discuss VHA parameters, interpretation, and goal-directed blood components and hemostatic adjuncts prior to discussing specific etiologies of SHINE shown in Figure 5.

## 3 Basic principles of VHAs

### 3.1 VHA tracing and parameters

**FIGURE 6:**
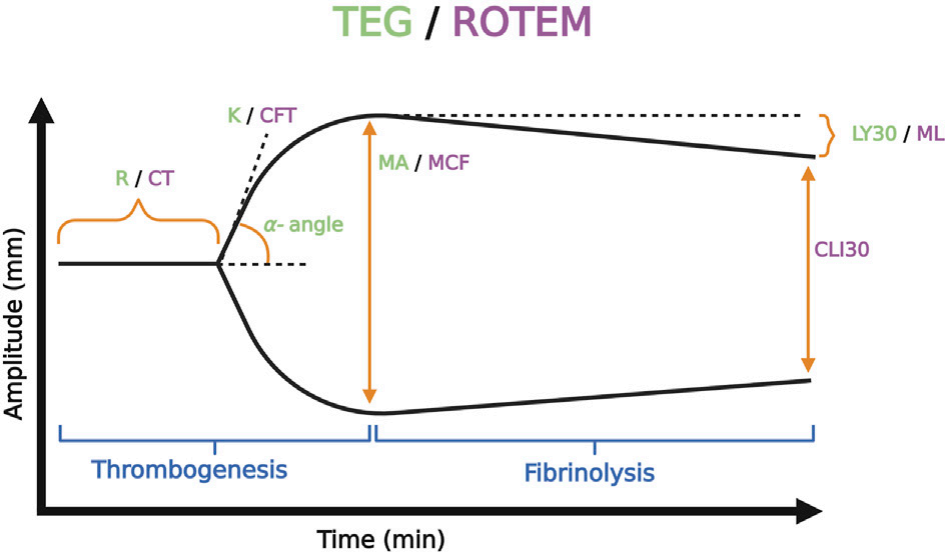
Representative Normocoagulable Thromboelastography (TEG) and Rotational Thromboelastometry (ROTEM) Tracing with Their Respective Parameters Defined. TEG and ROTEM parameters are represented by green and purple text, respectively. The time for the clot to reach 2 mm amplitude on the *y*-axis describes the reaction time (R) for TEG and clotting time (CT) for ROTEM. R and CT correlate to the activated partial thromboplastin time (aPTT) and prothrombin time (PT). The time spanned from 2 to 20 mm amplitude is called the kinetics (K) for TEG and the clot formation time (CFT) for ROTEM; these represent the speed of fibrin buildup. Likewise, alpha-angle measures the rate of fibrin buildup. The maximum amplitude (MA) on TEG and the maximum clot firmness (MCF) on ROTEM reflect crosslinking of fibrin with platelets and correspond to maximum clot retraction strength. Measurements of fibrinolysis include lysis at 30/60 min (LY30/60) which is the percentage decrease from MA achieved at 30/60 min, clot lysis index at 30/60 min (CLI30/60) which is the percentage of clot amplitude remaining relative to the MCF at 30/60 min, and maximum lysis (ML) which is the percentage decrease in MCF at a given length of time (Görlinger et al., 2021; Hartmann and Sikorski, 2021; Volod et al., 2022). Created with BioRender.com.

The mechanics of the cup-and-pin legacy devices (TEG 5000 and ROTEM delta), as well as the Sonic Estimation of Elasticity *via* Resonance (SEER) technology with SonoClot, and the new generation cartridge-based devices (i.e., ClotPro, Quantra, ROTEM Sigma, and TEG 6s) output tracings that plot the amplitude of clot strength in millimeters on the *y*-axis *versus* time in minutes on the *x*-axis. These tracings evaluate whole blood hemostatic competence by describing clot initiation, amplification, propagation, and termination by fibrinolysis (Curry et al., 2018; Volod et al., 2022). The maximum amplitude (MA) on TEG and maximum clot firmness (MCF) on ROTEM represent the surrogate endpoint of thrombogenesis. MA/MCF correspond to the maximal platelet-fibrin clot contraction strength; declining amplitude following the MA/MCF denotes fibrinolysis. TEG and ROTEM use differing reagents and parameter terminology. However, recognizing the similar pattern between the output tracing of the two tests allows for a broad comparison between the two devices. The differing terminology of the TEG/ROTEM, as well as other VHAs, has been viewed as a barrier to widespread clinical adoption. Figure 6 exemplifies the typical normocoagulable TEG/ROTEM tracing with the respective parameters defined. (Kang et al., 1985; Shore-Lesserson et al., 1999; Luddington, 2005; Spalding et al., 2007; Trzebicki et al., 2010; Sankarankutty et al., 2012; Whiting and DiNardo, 2014; Hunt et al., 2015; Gurbel et al., 2016; Veigas et al., 2016; Curry et al., 2018; Snegovskikh et al., 2018; Field et al., 2019; Gillissen et al., 2019; Schenk et al., 2019; Hartmann et al., 2020; Volod et al., 2022).

### 3.2 The shovel analogy to simplify VHA interpretation

**FIGURE 7:**
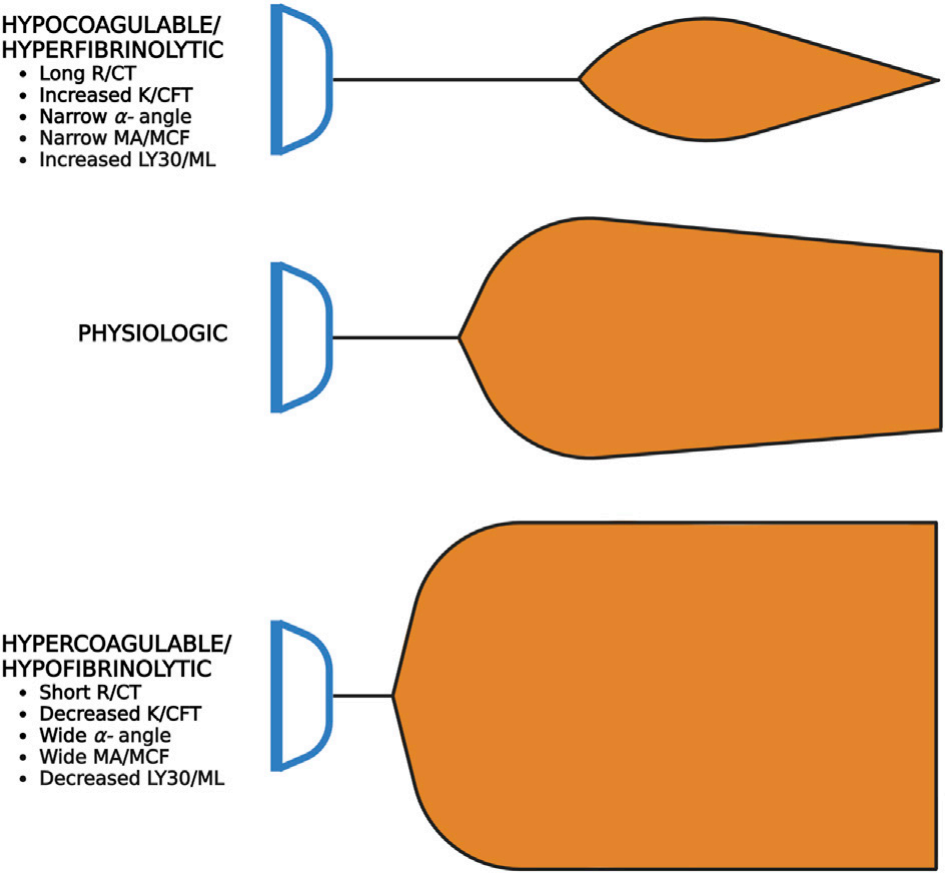
Shovel Analogy to Rapidly Interpret TEG/ROTEM Tracings. The top shovel represents the hypocoagulable state marked by a prolonged R/CT, narrow α-angle, narrow MA/MCF, and increased lysis with resultant increased LY30/ML. The middle shovel represents physiologic hemostasis marked by normal R/CT, α-angle, MA/MCF, and LY30/ML. Mild narrowing after the MA demonstrates physiologic fibrinolysis. The bottom shovel represents the hypercoagulable state denoted by decreased R/CT, wide α-angle, wide MA/MCF, and decreased LY30/ML. Abbreviations: R, Reaction time; CT, Clotting Time; K, Kinetics; CFT, Clot Formation Time; MA, Maximum Amplitude; MCF, Maximum Clot Firmness; LY30/60, Lysis at 30/60 min; ML, Maximum Lysis. Created with BioRender.com.

A useful analogy to simplify TEG/ROTEM interpretation embodies the tracing as the shape of a shovel (Figure 7). With hemostatic competence or physiologic hemostasis, the shovel has an ideal handle length (R/CT), blade slope (K/CFT and α-angle), blade width (MA/MCF), and blade tip (LY30/CLI30/ML), shown as the middle shovel in Figure 6. The extremes are represented by different shovel shapes where, for the sake of analogy, the ease of tilling and moving soil corresponds to the ease of moving blood. The hypocoagulable shovel tracing has a long handle with a narrow and pointed blade (top shovel in Figure 6), with which tilling and soil transport becomes less cumbersome but markedly inefficient. The hypercoagulable shovel tracing has a short handle and a wide blade with an absent tapering of the tip (bottom shovel in Figure 6), making it difficult for the earth to be broken up and transported. In the above examples, tilling and soil transport are either difficult (hypercoagulable) or easy but inefficient (hypocoagulable). In summary, in Figure 6, the top shovel represents hypocoagulable disequilibrium, the middle shovel represents physiologic hemostasis, and the bottom shovel represents hypercoagulable disequilibrium. Goal-directed BCT and HAT may be administered based on either the shovel analogy pattern or the numerical values of the TEG/ROTEM parameters (Table 2).

**TABLE 2:** Goal-directed blood components and hemostatic adjuncts based on the shovel analogy of TEG/ROTEM.

Parameters	Shovel analogy	Intervention
TEG: Reaction Time (R)	Long handle	Plasma and/or factor concentrates
ROTEM: Clotting Time (CT)	Short handle	Anticoagulation
TEG: kinetics (K); α-angle	Decreased slope of blade	Fibrinogen concentrate or cryoprecipitate
ROTEM: clot formation time (CFT); alpha angle		
TEG: Maximum Amplitude (MA)	Decreased width of blade	Platelets and/or fibrinogen concentrate/cryoprecipitate
ROTEM: Maximum Clot Firmness (MCF)	Increased width of blade	Anticoagulation and/or antiplatelet therapy
TEG: Lysis at 60 min (LY60)	Sharp taper of blade	Anti-fibrinolytics
ROTEM: Maximum Lysis (ML)/Clot Lysis Index at 60 min (CLI60)		

Abbreviations: CLI30/CLI60, Clot lysis index at 30/60 min; CT, clotting time; LY30, lysis at 30 min; MA, maximum amplitude; R, reaction time; ROTEM, rotational thromboelastometry; TEG, thromboelastography (Mamczak et al., 2022).

## 4 VHAs and etiologies of SHINE

### 4.1 Trauma-induced coagulopathy (TIC)

Uncontrolled hemorrhage accounts for about 25% of deaths after injury, and an estimated one-quarter of these deaths likely have a TIC element (Moore et al., 2021b). TIC is not a single entity, but rather comprises a spectrum of coagulopathic phenotypes that is largely biphasic. ‘Early TIC’ generally characterizes the first 6 h following injury wherein difficulty to achieve hemostasis may lead to hemostasis exhaustion, uncontrolled hemorrhage despite adequate mechanical control of bleeding sites (i.e., coagulopathy), and progressive hemorrhagic shock (Kashuk et al., 1982; Moore et al., 2021a). ‘Late TIC’ generally describes hypercoagulability 24 h or more following the time of injury. Clinically, late TIC manifests micro- and macro-thrombotic complications such as venous thromboemboli, ultimately leading to organ failure (Moore et al., 2021a). Early TIC severity increases proportionally with the magnitude of injury severity, blood loss, and shock. Late TIC correlates to the degree of tissue injury (Moore et al., 2021a).

Following major trauma, the release of tPA from endothelial cells may be involved in the initial activation of fibrinolysis in response to a burst of thrombin and fibrin generation and sympathetic outflow. This fibrinolytic phase ends within several hours by the production of PAI-1 by endothelial cells and platelets. This dynamic change is termed “fibrinolytic shutdown” (Moore et al., 2019b) and may rapidly occur in 40%–50% of patients despite arrival to the hospital within an hour after injury (Moore et al., 2016b). Hemorrhage may invoke physiologic fibrinolysis shutdown to achieve hemostasis at bleeding sites. However, trauma patients with persistent fibrinolytic shutdown at 24 h post-injury have increased mortality (Moore et al., 2019b). On the opposite end of the fibrinolytic spectrum, roughly one-quarter of trauma patients have evidence of prior fibrinolytic activation, but only 7% have active ongoing fibrinolysis at the time of initial blood draw (Moore et al., 2019a). Hyperfibrinolysis as measured by TEG/ROTEM correlates to increasing injury severity, magnitude of shock, catecholamines, and SHINE (Holcomb, 2011; Moore et al., 2016a). Administering TXA empirically to TIC patients without evidence of hyperfibrinolysis may cause early fibrinolysis resistance and increased mortality, necessitating a PBM approach to TXA use guided by VHAs (Moore et al., 2017).

**FIGURE 8:**
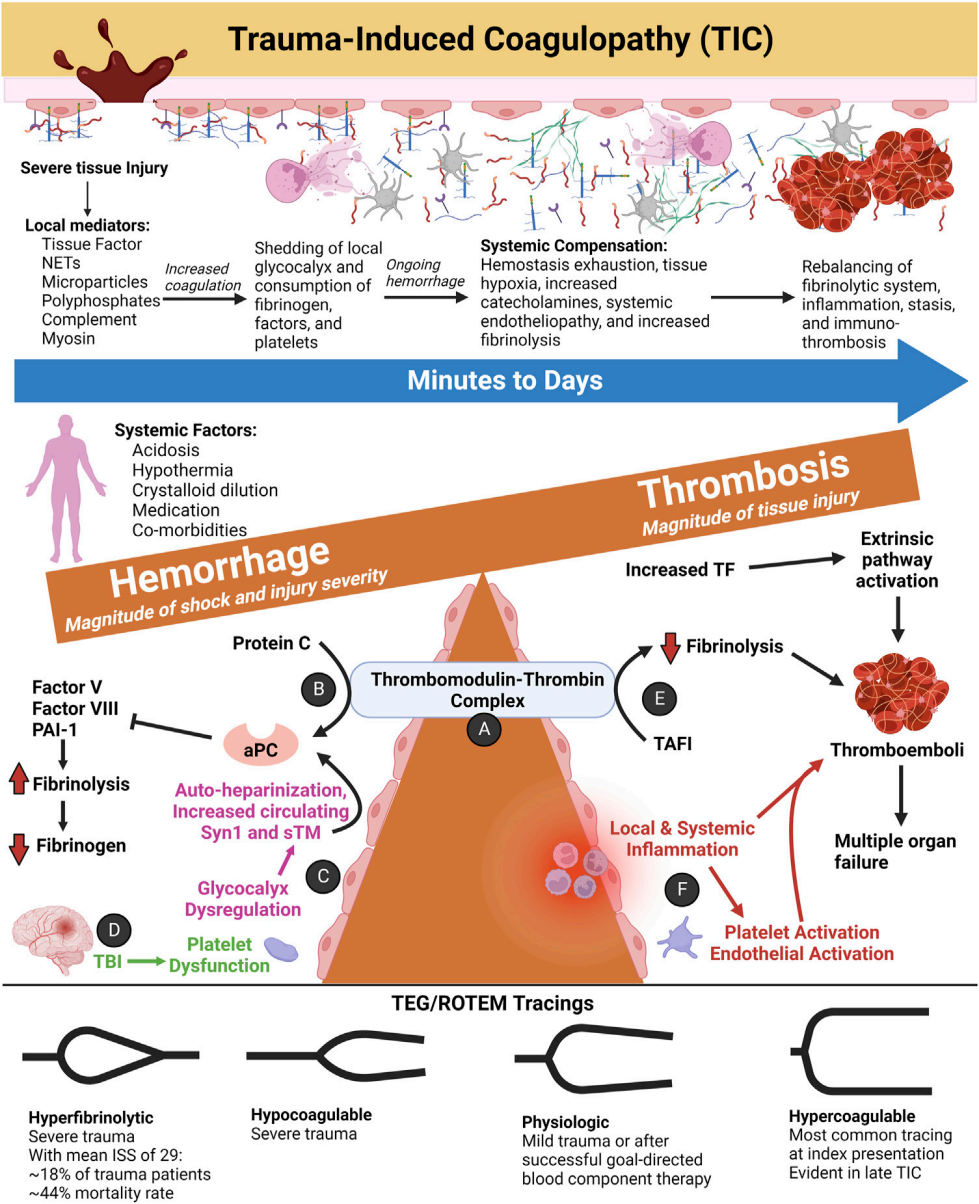
The Spectrum of Trauma-Induced Coagulopathy (TIC) as a Function of the Thrombomodulin-Thrombin Complex and SHINE. Hypercoagulability presents most commonly at index trauma presentation according to thromboelastography (TEG) and rotational thromboelastometry (ROTEM) tracings (Johansson and Ostrowski, 2010). As injury severity and the magnitude of hemorrhagic shock increase, the likelihood of hypocoagulability and/or hyperfibrinolysis increases in tandem (Johansson and Ostrowski, 2010; Moore et al., 2016a). Other anti-hemostatic factors at index may include acidosis, hypothermia, crystalloid resuscitation resulting in dilutional coagulopathy, pre-trauma anticoagulant or antiplatelet medications, and co-morbidities (Moore et al., 2021a). After successful initial resuscitation, patients most often demonstrate hypercoagulability and venous thromboembolism in the ensuing days. On the other hand, persistent fibrinolytic shutdown at 24 h post-injury correlates greatest to the magnitude of tissue injury. **(A)** The thrombomodulin (TM)-thrombin complex is one proposed hypothesis to explain TIC hemostatic phenotypes (Walsh et al., 2019). **(B)** In its anticoagulant role, the endothelial membrane-bound TM binds with thrombin to convert protein C to activated protein C (aPC). TM-thrombin action on protein C may also be accelerated by endothelial protein C receptor (EPCR, not shown). APC inactivates Factor V, Factor VIII, and plasminogen activator inhibitor-1 (PAI-1) to decrease coagulation and promote tissue plasminogen activator (tPA) activity to convert plasminogen to plasmin (Gando et al., 2018). The resulting fibrinolysis leads to hypofibrinogenemia and a hypocoagulable state as demonstrated by viscoelastic markers. APC and fibrinogen levels share an inverse relationship whereby the TM-thrombin complex increases protein C activation with decreasing fibrinogen levels, leading to a greater anticoagulant and pro-fibrinolytic state (Diez et al., 2006). On the contrary, with increased fibrinogen, the TM–thrombin complex is inhibited from activating protein C. As a result of glycocalyx dysfunction, activation of protein C, enhanced fibrinolysis, and low fibrinogen, the maladaptive response caused by consumption of clotting factors and platelets leads to high fibrin/fibrinogen degradation products (FDPs) with an overall anti-hemostatic state (Diez et al., 2006; Dobson et al., 2015). **(C)** Tissue hypoperfusion and endothelial injury causes shedding of the endogenous HS of the glycocalyx with subsequent “auto-heparinization” (Ostrowski and Johansson, 2012). The sensitivity of TEG/ROTEM to detect auto-heparinization remains questionable (Zipperle et al., 2022a). Disruption of the endothelial glycocalyx may also be measured by increased circulating syndecan-1 (Syn1) and soluble TM (sTM) levels (Johansson et al., 2011b). **(D)** Traumatic brain injury produces a unique coagulopathy characterized by platelet dysfunction at the arachidonic acid (AA) and adenosine diphosphate (ADP) receptors as defined by TEG with Platelet Mapping. The relatively high concentrations of von Willebrand Factor (vWF) and Tissue Factor (TF) release from injured brain tissue are thought to cause platelet exhaustion (Castellino et al., 2014; Bradbury et al., 2021). However, the pathophysiology of coagulopathy of traumatic brain injury remains an area of active study. **(E)** The TM-thrombin complex also activates thrombin-activatable fibrinolysis inhibitor (TAFI) which acts to inhibit tPA binding to fibrin (Marar and Boffa, 2016). **(F)** Minutes to days after traumatic/surgical-related injury, local and/or systemic inflammation occurs, causing immuno-thrombosis *via* platelet and endothelial activation. Particularly in the microvasculature, thromboemboli impair organ perfusion and contribute to organ failure (Gando and Otomo, 2015). Abbreviations: aPC, activated Protein C; ISS, Injury Severity Score; NETs, Neutrophil Extracellular Traps; PAI-1, Plasminogen Activator Inhibitor-1; ROTEM, Rotational Thromboelastometry; TAFI, Thrombin-Activatable Fibrinolysis Inhibitor; TEG, Thromboelastography; TF, Tissue Factor; TIC, Trauma-induced Coagulopathy; TBI, Traumatic Brain Injury. Created with BioRender.com.

Figure 8 depicts the coagulofibrinolytic balance of TIC as a teeter totter wherein the TM-thrombin complex is one such fulcrum to determine anti- or pro-hemostatic phenotypes. This complex, also mediated by the endothelial protein C receptor (EPCR), activates the aPC anticoagulation pathway resulting in Factor V and Factor VIII degradation and enhanced fibrinolysis *via* PAI-1 inhibition. On the contrary, the TM-thrombin complex may activate the TAFI pathway resulting in hypercoagulation by inhibiting fibrinolysis (Sillen and Declerck, 2021). Whether the action of the TM-thrombin complex favors increased coagulation *via* TAFI or a hypocoagulable state mediated by aPC on the activated endothelium depends on the severity of trauma, the presence or absence of shock, endotheliopathy, and the manner, timing, and response to resuscitation (Dobson et al., 2015; Moore et al., 2020). The balance of the two opposing pathways may tip directions within seconds to minutes following trauma and involves either structural and/or posttranslational modifications of different sites on the TM-thrombin complex. Subsequent action by receptors and/or cofactors causes the modified TM-thrombin complex to bind and activate protein C or TAFI (Dobson et al., 2015; Johansson et al., 2017a).

With vascular injury, a thrombin burst mediates fibrin formation as well as a protection of the fibrin clot from dissolution *via* activation of TAFI (Lord, 2011; Foley et al., 2013). tPA or uPA cleavage of plasminogen to plasmin, the major fibrinolytic enzyme, then dissolves the fibrin meshwork into soluble fibrin/fibrinogen degradation products (FDPs) which mediate a positive feedback mechanism resulting in fibrinolysis (Silva et al., 2012). PAI-1 primarily prevents hyperfibrinolysis by inhibition of tPA as well as urokinase plasminogen activator (uPA) (Declerck and Gils, 2013; Sillen and Declerck, 2020). In addition, plasmin is inhibited by α2-antiplasmin (Singh et al., 2020). Importantly, activated TAFI (TAFIa) is a zinc-dependent metallocarboxypeptidase which downregulates fibrinolysis by removing C-terminal lysine residues from partially degraded fibrin; thereby preventing the upregulation of plasminogen binding and activation (Declerck, 2011; Vercauteren et al., 2013). Activation of TAFI following the thrombin burst regulates hemostasis with a fibrinolytic shutdown response and has been described as a crucial regulatory link between coagulation and fibrinolysis (Leurs and Hendriks, 2005; Claesen et al., 2021).

Recent studies have demonstrated mortality benefit and cost savings associated with early plasma resuscitation for patients with TIC (Moore et al., 2018; Brill et al., 2021). Early administration of plasma may serve therapeutic and sparing effects on the endothelial glycocalyx layer as demonstrated by decreased Syn-1 levels following plasma administration (Cannon, 2018; Sperry et al., 2018; Gruen et al., 2020; Pusateri et al., 2020; Hrebinko et al., 2021). This reduction of Syn-1 shedding may occur *via* reduced Tissue Inhibitor of MetalloProteinase (TIMP) activity or decreased activation of A Disintegrin And Metalloproteinase (ADAM). VHAs have been recommended as a method to gauge the adequacy of targeted resuscitation with plasma (Moore et al., 2021a).

Emphasis on resuscitation of the endothelium has led to the use of vasopressin for patients in severe shock associated with trauma (Simmons and Powell, 2016). When used in conjunction with clinical, laboratory, biologic, and standard coagulation tests, adjunctive VHAs reflect the hemostatic milieu of the endothelium and its contribution to hemostatic derangement in patients with SHINE. The combination of these tests provides a holistic view of whole blood hemostatic integrity, enabling goal-directed plasma and/or pressor therapy for patients in hemorrhagic shock (Moore et al., 2021a; Richards et al., 2021).

SHINE and mortality in TIC correlate to blood product administration (Dunne et al., 2004). It has been suggested that the pro-inflammatory extracellular vesicles (EVs) in stored blood products, particularly packed red cells, may cause or contribute to endotheliopathy (Straat et al., 2016). However, a recent observational study of 75 trauma patients demonstrated that red blood cell EVs increased following transfusion yet did not increase Syn1 levels (Dujardin et al., 2022).

Trauma can also be classified as primary or secondary based on pathophysiology (Brohi et al., 2003; Simmons and Powell, 2016), early or late based on timing (Tisherman et al., 2015), hypo- or hyperfibrinolytic based on hemostatic phenotype, and resuscitated or not resuscitated (Gando et al., 1992; Gando et al., 1995; Adrie et al., 2004; Gando et al., 2011; Tauber et al., 2011; Wohlauer et al., 2012; Gando et al., 2013; Moore et al., 2015b; Vogel et al., 2015; Gando and Hayakawa, 2016; Davenport et al., 2017; Leeper et al., 2017; Macko et al., 2017; Meizoso et al., 2017). Without treatment, these patients may progress to a DIC-like syndrome of hyperfibrinolysis in minutes to hours. Therefore, point-of-care testing with VHAs enables hemostatic monitoring to guide diagnosis and individualized ratios of BCT and HATs (Moore et al., 2019b; Moore et al., 2020). It should also be noted that surgical-related coagulopathies, such as damage control surgery or those incurred during liver transplantation and cardiac surgeries which have a long and rooted history of VHA-guided BCT and HAT, may be viewed similarly to TIC. Surgical-related coagulopathies and TIC share traumatic hemorrhagic shock pathophysiology and likewise necessitate goal-directed resuscitation.

### 4.2 Sepsis-induced coagulopathy (SIC)

**FIGURE 9:**
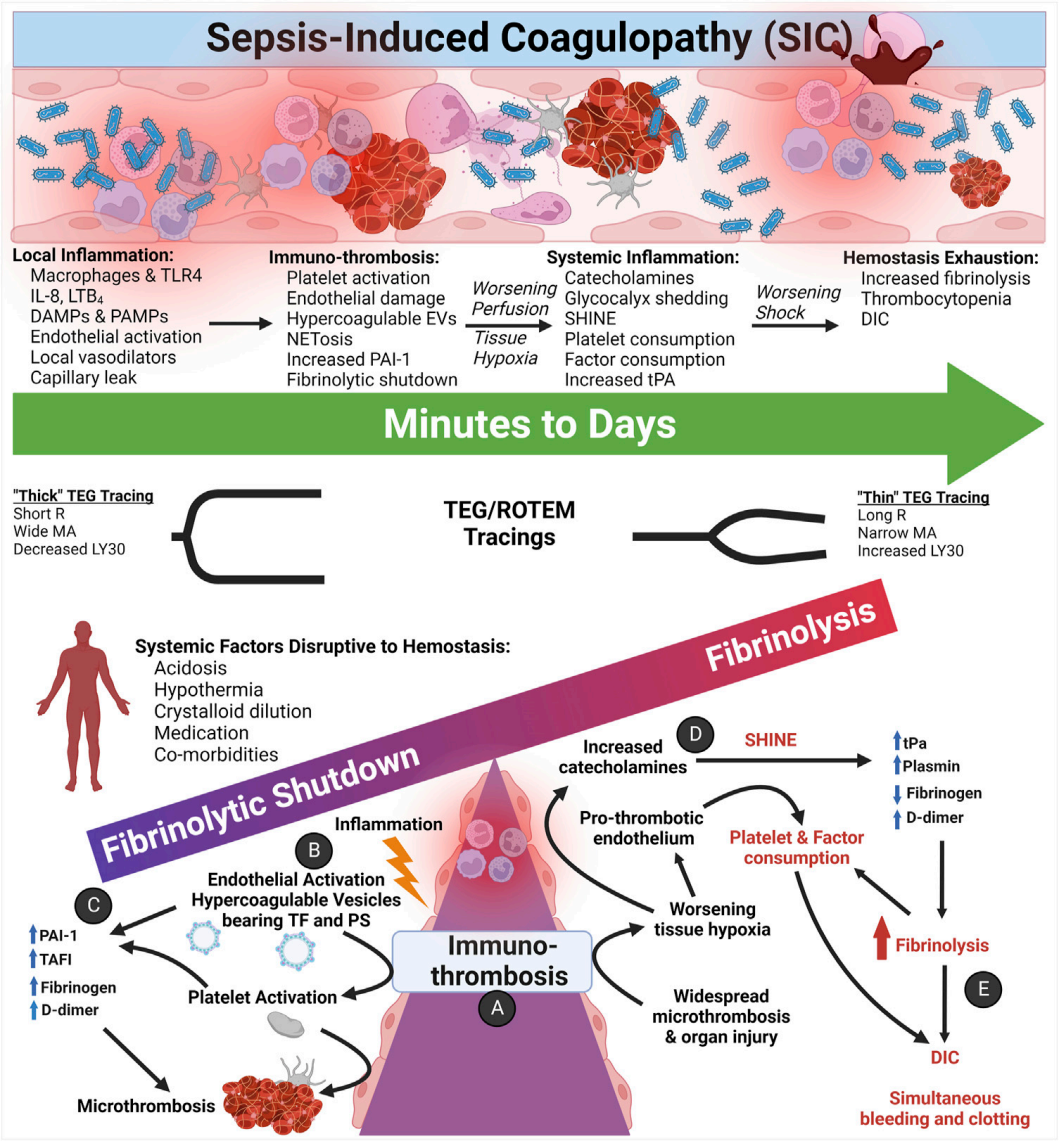
The Coagulofibrinolytic Spectrum of Sepsis-induced Coagulopathy (SIC) Pertaining to Immuno-thrombosis and SHINE. **(A)** Initially, the immuno-thrombosis manifests as microthrombosis within the microvasculature. **(B)** Inflammation activates the endothelium and, among other mechanisms, activates primary and secondary hemostasis *via* the endothelial release of hypercoagulable circulating extracellular vesicles (EVs) bearing Tissue Factor (TF) and phosphatidylserine (PS). **(C)** Most patients with SIC present with hypercoagulopathic, hypofibrinolytic thromboelastography (TEG)/rotational thromboelastometry (ROTEM) tracings with elevated acute phase reactants such as fibrinogen, D-dimer, and plasminogen activator inhibitor-1 (PAI-1). Quiescent platelets contain PAI-1, TAFI, FXIIIa, and α2-antiplasmin in α-granules, and upon activation, platelets release PAI-1 to complex with and inhibit action of tPA. Thrombin may also provoke release of PAI-1 from the endothelium (Huebner et al., 2018). **(D)** As hypoperfusion and the shock state progresses, increased catecholamines activate and damage the pro-thrombotic endothelium, causing systemic endothelial release of Weibel-Palade bodies containing tPA. Hypoperfusion also increases endothelial calcium influx, resulting in PS exposure on the endothelial luminal surface. **(E)** Increased circulating tPA tips the scales in favor of fibrinolysis as a counterbalance to the widespread microthrombosis. Thus, a small percentage of septic patients may present and/or progress to a hyperfibrinolytic and consumptive hypocoagulopathic state of disseminated intravascular coagulation (DIC), which requires aggressive resuscitation with primarily blood components as opposed to crystalloid fluids for the hypercoagulopathic SIC patients (Levi and van der Poll, 2017; Iba and Ogura, 2018; Iba et al., 2019b; Bunch et al., 2022b). Abbreviations: DAMPs, Damage-Associated Molecular Patterns; DIC, Disseminated Intravascular Coagulation; EVs, Extracellular Vesicles; IL-8, Interleukin-8; LTB4, Leukotriene B4; LY30, Lysis at 30 min; MA, Maximum Amplitude; PAI-1, Plasminogen Activator Inhibitor-1; PAMPs, Pathogen-Associated Molecular Patterns; PS, PhosphatidylSerine; R, Reaction time; SHINE, SHock-INduced-Endotheliopathy; TAFI, Thrombin-Activatable Fibrinolysis Inhibitor; TF, Tissue Factor; TLR, Toll-Like Receptors; tPA, tissue Plasminogen Activator. Created with BioRender.com.

Whereas hemorrhagic shock with TIC potentiates uncontrollable bleeding in its early phase, the early hemostatic phenotype of SIC involves a hypercoagulable state of hypofibrinolysis with consequent micro-thrombosis and sequential organ failure (Semeraro et al., 2012). Trauma and surgery patients who survive the early phase of hemorrhage may develop a late hemostatic phenotype that manifests as thrombosis and multiple organ failure like SIC. However, the stimulus of coagulation for each pathologic entity differs. In TIC, tissue factor (TF) release from injured tissue induces coagulation. Micro-thrombus formation, a fundamental event of SIC, is observed in late TIC and is under continued investigation (Vogel et al., 2015; Moore et al., 2020). In SIC, the two main drivers at the level of the endothelium are immuno-thrombosis and suppressed fibrinolysis. The influence of these systems and the mechanisms of their effect on the endothelium are depicted in Figure 9.

The mechanisms that initiate SIC have been previously described as both cell-based and humoral-based (Liaw et al., 2016; Iba and Levy, 2018). At the interface of mounting an immune response, the endothelium activates to a pro-thrombotic state in response to numerous inflammatory mediators. Biomarkers that crosstalk between inflammation and endothelial activation include leukotrienes, IL-1, IL-6, IL-8, TNF-α, reactive oxygen species, hydrogen peroxide, complement, histamine, serotonin, and shiga toxin, as well as hypoxia, thrombin, fibrin, and epinephrine (McCormack et al., 2017). The host response to sepsis involves the activation of coagulation by TF on EVs and activated endothelium (Østerud and Bjørklid, 2001). PS expressed on EVs and activated endothelium also activates the extrinsic coagulation cascade (Iba and Ogura, 2018). Among the most salient factors which are involved in the immuno-thrombotic response to sepsis are pathogen-associated molecular patterns (PAMPs), damage-associated molecular patterns (DAMPs), high mobility group box 1 (HMGB 1), DNA, histones, neutrophil extracellular traps (NETs), damaged host cells, and activated immune cells, all of which initiate pro-inflammatory and pro-thrombotic reactions in SIC (Østerud and Bjørklid, 2001; Brinkmann et al., 2004; Adrie et al., 2005; Liaw et al., 2016; Iba and Ogura, 2018; Vulliamy et al., 2019).

FDPs and D-dimer levels have limited use for diagnosing and treating shock in either SIC of TIC. Because of their long half-life these markers do not correlate with PAI-I levels in patients with SIC or TIC. PAI-I levels are not readily available in clinical practice and therefore VHAs have been used to detect fibrinolysis in trauma patients. (Moore et al., 2014). Despite widespread use of VHA to detect fibrinolysis in trauma there is significant debate regarding its sensitivity (Larsen et al., 2012; Hunt et al., 2013; Ramos et al., 2013; Raza et al., 2013; Stettler et al., 2019).

AT is an important anticoagulant that prevents the formation of thrombi (Levy et al., 2016). In addition, prostacyclin, nitric oxide, and TFPI mediate anti-thrombotic effects at the level of the endothelium (Iba and Levy, 2019).

In SIC there is a significant suppression of anti-thrombotic activity which is affected by the methods of treatment as well as the speed of resuscitation. CCTs as well as FDPs and D-dimers do not adequately assay the importance of anti-thrombotic activity in SIC and TIC (Owings et al., 1996; Iba et al., 2012). It is instructive to compare early TIC and late TIC with SIC, whereby the increased release of TM activates protein C whereas late in TIC AT and protein C are depressed (Hess et al., 2008; Zilkens et al., 2008; Yanagida et al., 2013; Choi et al., 2014; Johansson et al., 2017a; Kornblith et al., 2019; Keshari et al., 2020). In sepsis AT levels decline and recent studies have demonstrated the possible utility of AT therapy in septic DIC (Vincent et al., 2019; Egi et al., 2021).

The similarities between SIC and TIC are instructive and are summarized in Table 3. Whereas the hemostatic derangement characteristic of hyperfibrinolytic phenotype associated with early severe TIC in shock is transformed in late TIC into a hypofibrinolytic phenotype characteristic of SIC (Taylor Jr et al., 2001; Gando and Wada, 2019; Vulliamy et al., 2019; Moore, 2022).

**TABLE 3:** Contrasting hemostatic changes in TIC and SIC.

	Early-TIC	Late-TIC	SIC
Coagulation	activated	activated	activated
Anticoagulation	usually absent	impaired	impaired
Fibrinolysis	increases with injury severity	suppressed	suppressed
Platelet function	activated	activated	activated
Endothelium/glycocalyx	damaged and contributes to anticoagulation	damaged and becomes pro-thrombotic	damaged and becomes pro-thrombotic
Micro-thrombus	usually absent	present	present
Phenotype	bleeding-dominant	pro-thrombotic and develops organ dysfunction	pro-thrombotic and develops organ dysfunction

Abbreviations: SIC, Sepsis-induced coagulopathy; TIC, trauma-induced coagulopathy.

### 4.3 Post-cardiac arrest syndrome (PCAS)-associated coagulopathy

**FIGURE 10:**
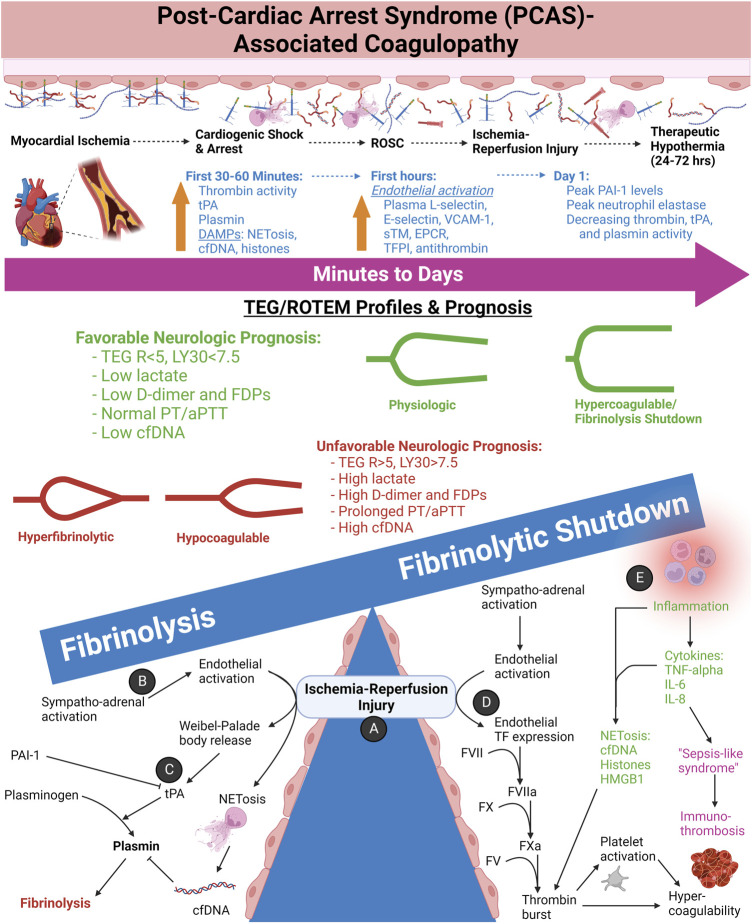
The Spectrum of Post-Cardiac Arrest Syndrome (PCAS)-associated Coagulopathies and Neurologic Prognostication by TEG/ROTEM. **(A)** In cardiac arrest, ischemia afflicts every tissue in the body. Depending on the length of arrest, necrosis results for many tissue types, resulting in an acute inflammatory response. Return of spontaneous circulation (ROSC) further promotes inflammation by reperfusion of oxygen, thereby increasing the generation of reactive oxygen species by the now resident inflammatory cells. **(B)** As a result of the shock state and epinephrine infusion during resuscitation, the activated endothelium becomes pro-thrombotic and simultaneously fibrinolytic *via* Weibel-Palade body (WPB) exocytosis as one such mechanism. **(C)** Widespread release of tissue plasminogen activator (tPA) by the endothelium promotes conversion of plasminogen to plasmin. Circulating cell free DNA (cfDNA), either from neutrophil extracellular traps (NETs) or necrotic cells, has demonstrated to inhibit plasmin activity to a degree. Circulating plasminogen activator inhibitor-1 (PAI-1) also serves to decrease fibrinolytic activity; however, as an acute phase reactant, PAI-1 levels have shown to peak at 24 h following ROSC. Platelet activation and release of α-granule contents PAI-1, TAFI, FXIIIa, and α2-antiplasmin likely also contribute. Hyperfibrinolysis and/or hypocoagulability prognosticate poor neurologic outcomes. These hemostatic phenotypes arise more commonly with longer times to achieve ROSC. TEG measurements of reaction time (R) > 5 min and lysis at 30 min (LY30) >7.5% following ROSC tend to have poor neurologic outcomes. In tandem, prolonged prothrombin time (PT) and activated partial thromboplastin time (aPTT) and increased markers of fibrinolysis (e.g., D-dimer and fibrin [ogen] degradation products) also prognosticate poor outcomes. Increased markers of tissue ischemia and necrosis such as lactate and cfDNA follow a similar worse prognosis. **(D)** Endothelial activation promotes thrombosis by increased Tissue Factor (TF) expression by both increased extracellular vesicles bearing TF, but also by necrotic cells releasing free TF systemically. **(E)** The ensuing inflammatory state in response to ischemia promotes immuno-thrombosis *via* several mechanisms, but namely *via* NETs catching and activation of circulating platelets as well as pro-thrombotic proteins from necrotic tissues such as cfDNA, histones, and High Mobility Group Box-1 (HMGB-1). The inflammatory state observed clinically in PCAS patients has been aptly termed “Sepsis-like syndrome” because of the systemic inflammatory response syndrome without an infectious source (Wada, 2017; Yu et al., 2020). Important to note, however, that hyperfibrinolysis in PCAS appears to be caused primarily by hypoperfusion rather than inflammation (Zipperle et al., 2022b). Abbreviations: aPTT, activated Partial Thromboplastin Time; cfDNA, cell free DNA; DAMPs, Damage-Associated Molecular Patterns; EPCR, endothelial Protein C Receptor; FDPs, Fibrin(ogen) degradation products; HMGB-1, High Mobility Group Box 1; IL, Interleukin; LY30, Lysis at 30 min; NETs, Neutrophil Extracellular Traps; PAI-1, Plasminogen Activator Inhibitor-1; PT, Prothrombin Time; R, Reaction time; sTM, soluble Thrombomodulin; TFPI, Tissue Factor Pathway Inhibitor; TNF-alpha, Tissue Necrosis Factor-alpha; tPA, tissue Plasminogen Activator; VCAM-1, Vascular Cellular Adhesion Molecule-1. Created with BioRender.com.

After TIC and SIC, the third most common cause of shock is cardiogenic of which PCAS is a major subtype. Ischemia-reperfusion injury drives the pathophysiology of PCAS-associated coagulopathy. The ensuing tissue necrosis, pro-thrombotic DAMPs, systemic inflammatory response, sympatho-adrenal activation, and SHINE account for the commonly observed hypercoagulability in these patients. The hypercoagulability arises from increased circulating TF, DAMPs, immuno-thrombosis, and an activated pro-thrombotic endothelium. Acutely, systemic hyperfibrinolysis occurs in PCAS because of tPA release from endothelial Weibel-Palade bodies. Hyperfibrinolysis occurs in about one-third to one-half of patients with PCAS, confirming the high incidence of fibrinolysis in patients with the “no-reflow phenomenon” of PCAS (Ames et al., 1968; Schöchl et al., 2013; Kloner et al., 2018). Of note, a recent observational study of 41 patients with cardiac arrest has supported conventional activation of plasminogen—as opposed to pro-inflammatory pathways of fibrinolysis activation—as the cause of hyperfibrinolysis in PCAS-associated coagulopathy (Zipperle et al., 2022b). This study also supported that hyperfibrinolysis in PCAS shares pathophysiologic similarities to TIC wherein hypoperfusion and increased aPC appear to be the incipient drivers. Moreover, it has been observed that cardiac arrest due to hypoxia has a higher incidence of hyperfibrinolysis compared to cardiac arrest from a primarily cardiogenic cause (Wada et al., 2017). Figure 10 depicts the coagulofibrinolytic equilibrium of PCAS-associated coagulopathies wherein ischemia-reperfusion injury determines the balance between hyperfibrinolytic and fibrinolysis shutdown phenotypes.

Compared to patients without the hyperfibrinolytic phenotype, patients with hyperfibrinolysis required longer CPR times, had elevated aPTT, D-dimer, and hypoperfusion markers including pH, base excess, and lactate. The lysis onset time (LOT) was directly proportional to survival and inversely related to CPR times and lactate. These data confirmed previous observations that the time to onset of clot lysis is an important marker for patient outcomes (Viersen et al., 2012). High lactate levels also predict development of PCAS-associated DIC with hyperfibrinolysis (Wada et al., 2016).

On the contrary, small increases in PAI-1 levels are measurable shortly after ROSC and may be owed to release by activated platelets or endothelium. PAI-1 levels have been shown to peak at 24 h after achieving ROSC, and increased levels correlate to multiple organ dysfunction and worse outcomes (Geppert et al., 2001; Wada, 2017). The initial hypercoagulopathic phase in patients with PCAS reflect similarly to severe TIC with early phase hyperfibrinolysis mediated by tPA and subsequent fibrinolytic shutdown mediated by PAI-1. The unique pathophysiologic moment begins with the “no-reflow phenomenon” which describes reduced antegrade coronary and/or cerebral microcirculatory blood flow despite proximal patency which is commonly seen following cardiac arrest and ROSC (Ames et al., 1968; Kloner et al., 2018). The rapid change from hyperfibrinolysis to hypofibrinolysis occurs with successful and early ROSC. Unlike TIC, these patients do not benefit from anti-fibrinolytic administration which substantiates the lack of similar causality for the coagulopathies associated with PCAS (Ames et al., 1968; Wada, 2017; Yu et al., 2020).

Studies of patients with PCAS who have attained ROSC have demonstrated the utility of TEG and ROTEM to predict intact neurologic survival as a reflection of reduced fibrinolysis. It has been shown that TEG values of R < 5 min or LY30 < 7.5% in early PCAS had more favorable neurologic outcomes. Higher D-dimer levels, PT, aPTT, lactate, and cfDNA were noted in the unfavorable outcome group. Therefore, in the earliest periods following ROSC, a normal hemostatic and fibrinolytic phenotype are early predictors of neurologically intact survival in successfully resuscitated out-of-hospital cardiac arrest patients (Yu et al., 2020). Early ROTEM analysis has likewise revealed a high incidence of hyperfibrinolysis for those patients who had long cardiac arrest times and poor prognosis. Specifically, hyperfibrinolysis criteria have been recorded in 83% of patients with long cardiac arrest times, and these patients also had lower fibrinogen levels with corresponding low levels of FIBTEM MCF (Barea-Mendoza et al., 2019).

The use of mild therapeutic hypothermia (MTH) has demonstrated increased survival. TEG may be a useful technique to evaluate hemostatic integrity in cardiac arrest survivors undergoing MTH. However, the effects of MTH on PCAS-associated coagulopathy requires appreciation for the effect of temperature on fibrin (ogen) concentration and function. When compared to physiologic temperature patients who have survived cardiac arrest, MTH has shown to lengthen TEG R, reduce the coagulation index (CI), and attenuate clot fibrinolysis. Rather than performing the VHAs at 37°C, it is therefore suggested that VHA analysis be performed at 32°C during MTH to increase the accuracy of hypothermic coagulation impairment (Trąbka-Zawicki et al., 2019). Moreover, prolonged MTH has shown to impair thrombin generation as measured by increased CT and prolonged time to maximum velocity of thrombin generation on INTEM (Jeppesen et al., 2017).

### 4.4 Medical non-obstetrical hemorrhage

Common non-obstetrical causes of shock include gastrointestinal (GI) hemorrhage and, to a lesser extent, retroperitoneal hemorrhage of anticoagulated or hypocoagulopathic patients. The state of the endothelium in many ways reflects TIC where the degree of hemorrhage dictates the hemostatic derangement at the endothelium. However, as in TIC, pre-existing hemostatic phenotypes (e.g., liver failure and antiplatelet or anticoagulant medications) in part determine the evolution and response to therapy which requires VHAs to guide BCT and HAT for these patients (Bunch et al., 2022a). Patients treated with anticoagulants and antiplatelet agents often require replenishment of factors and/or platelets in TIC and medical hemorrhage causing shock (Kang et al., 1985; Shore-Lesserson et al., 1999; Enriquez and Shore-Lesserson, 2009; Ojito et al., 2012; Tanaka et al., 2012; Levin et al., 2014; Tanaka et al., 2014; Weitz and Eikelboom, 2016; Bliden et al., 2017; Douketis et al., 2017; Dubois et al., 2017; Gurbel et al., 2017; Mullins et al., 2018; Artang et al., 2019; Bruckbauer et al., 2019; Dias et al., 2019; Pailleret et al., 2019; Sarode, 2019; Oberladstätter et al., 2021; Pavoni et al., 2022). In patients with liver failure and GI hemorrhage, the rebalanced hemostasis caused by the reduced anticoagulants protein S, protein C, and AT require that VHAs be used in the diagnosis and resuscitation of these patients in shock. Serial hemostatic functional evaluation of a patient in liver failure with shock would not be possible with CCTs, but can be done successfully with VHAs (Groth et al., 1969; Chau et al., 1998; Starzl, 2002; Tripodi and Mannucci, 2011; Agarwal et al., 2012; Stravitz, 2012; Scarlatescu et al., 2018; Stravitz et al., 2021). Interestingly, the use of anti-fibrinolytics in patients with GI hemorrhage and shock has not shown to improve outcomes, further demonstrating the heterogeneity of coagulopathies associated with SHINE due to medical hemorrhage *versus* traumatic or surgery-related hemorrhage (Roberts et al., 2020).

### 4.5 Postartum hemorrhage (PPH)

VHA-guided BCT during PPH is expanding. TEG/ROTEM devices can be used to detect and treat clinically significant hypofibrinogenemia, although evidence to support the role of VHAs for guiding fresh frozen plasma and platelet transfusion is less clear (Collis and Collins, 2015; Bamber, 2016; Collis, 2016; Curry et al., 2018). If ROTEM/TEG tracings are normal, clinicians should investigate for another cause of bleeding, and BCT may be withheld. Guidelines support the use of VHAs during PPH if a local algorithm reaches agreement. However, wide consensus does advise that a FIBTEM amplitude at 5 min (A5) of <12 mm with ongoing bleeding necessitates fibrinogen replenishment (Curry et al., 2018; Collins, 2022). Note, however, that patients with PPH may also have reduced thrombin generation (Di Bartolomeo et al., 2017). Guidelines recommend against using VHAs to guide TXA infusion. Rather, TXA should be administered as soon as PPH is diagnosed irrespective of the TEG/ROTEM traces, however patients with PPH are also at high for venous thromboembolism suggesting this issue requires further investigation (Shakur et al., 2010; Roberts et al., 2012; Collins, 2022). The cost-effectiveness of VHAs during PPH needs to be addressed and has formed the foundation for much of the discussion regarding the utility of VHAs to guide BCTs and HATs in patients with severe PPH. (Collis and Collins, 2015; Bamber, 2016; Collis, 2016; Curry et al., 2018; Collins et al., 2019; Liew-Spilger et al., 2021; Collins, 2022).

Severe PPH, such as with amniotic fluid embolism (AFE), often associates with hyperfibrinolysis like severe TIC. However, the etiology and treatment differ significantly from severe TIC and requires VHA-guided resuscitation. The mechanism for AFE-induced coagulopathy is the release of amniotic fluid TF into the systemic and pulmonary circulation, provoking a classic DIC of overwhelming thrombin generation, consumption of clotting factors and platelets, and hyperfibrinolysis (Harnett et al., 2005; Bassily-Marcus et al., 2012).

Treatment of AFE entails the immediate delivery of the fetus, VHA-guided resuscitation of the mother, and emphasis for early administration of fibrinogen to levels that are two to three times higher than normal to outcompete platelet and clotting factor consumption, and fibrinolysis (Kramer et al., 2013; Dahlke et al., 2015; Guasch and Gilsanz, 2016; Pacheco et al., 2016; Henriquez et al., 2018; McDonnell and Browning, 2018; Amgalan et al., 2020; Liew-Spilger et al., 2021). Early PPH not associated with AFE may be diagnosed with modified VHAs designed to detect low levels of fibrinogen, which when properly treated, enable reduced incidence of shock and BCT use in women who suffer PPH (McNamara et al., 2015; Collins et al., 2017; Loughran et al., 2019; Othman et al., 2019; Liew-Spilger et al., 2021). In addition, an endogenous heparin-like substance has been associated with the coagulopathy of severe PPH and subsequent shock as identified by TEG (Wang et al., 2020). The placenta also contains high levels of TFPI (Kuczyński et al., 2002; Xiong et al., 2010).

As with late TIC and SIC as well as late PCAS, fibrinolytic shutdown post-resuscitation of PPH remains a risk factor for venous thromboembolism (VTE). Therefore, VHAs may help guide the intensity and duration of DVT prophylaxis in postpartum women, particularly those who suffered hemorrhagic shock (Maybury et al., 2008).

**FIGURE 11:**
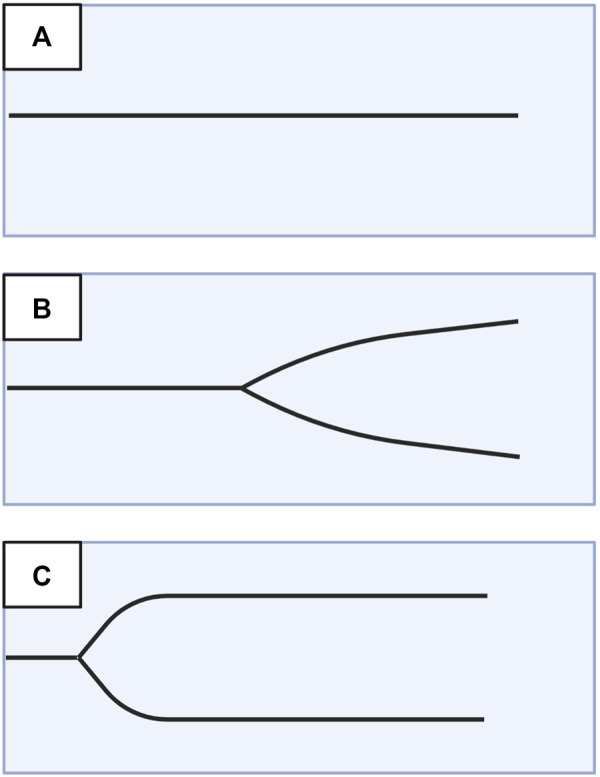
Evolution of Thromboelastography (TEG) Tracings During Resuscitation of a Patient with Amniotic Fluid Embolism. During induction of labor, a 35-year-old woman had a sudden cardiac arrest due to amniotic fluid embolism (AFE). She developed immediate disseminated intravascular coagulation, respiratory failure, and renal failure requiring mechanical ventilation and dialysis. Immediate delivery of the fetus by cesarean section was followed by normal APGAR scores at 9 min. Both child and mother were discharged from the hospital with no residual complications. TEG tracing **(A)** demonstrates a flat line indicating no clot formation. Two hours after the first blood draw, the laboratory called to say that the aPTT was excessively prolonged and must be a laboratory error. TEG tracing **(B,C)** show gradual improvement of TEG tracings at 2 and 8 h following cardiac arrest. Resuscitation required in total 12 units of packed red blood cells, six units of plasma, three units of platelets, four 10-unit doses of cryoprecipitate, two doses of recombinant factor VIIa at 80 μg/kg/dose, and 2,000 units of prothrombin complex concentrate (Hurwich et al., 2016). Abbreviations: aPTT, activated Partial Thromboplastin Time; TEG, Thromboelastography. Created with BioRender.com.

Figure 11 exemplifies VHA-guided resuscitation for a 35-year-old woman with cardiac arrest due to AFE (Hurwich et al., 2016). Hemostatic management of the obstetrical patient with this severe type of SHINE remains difficult without bedside adjunctive VHAs, whether due to AFE or other causes of PPH such as eclampsia, hemolysis with elevated liver enzymes and low platelets (HELLP Syndrome), or structural anomalies with and without uterine atony (Amorde et al., 2011; Loughran et al., 2019).

### 4.6 Hyperthermia/burns and non-therapeutic environmental hypothermia

Severe hyperthermia, which causes burn-induced coagulopathy (BIC), can present with early hyperfibrinolysis and hypocoagulability. With treatment of shock, these patients most often develop a hypofibrinolytic and hypercoagulable state as manifested by VHAs (Marsden et al., 2017; Borgman et al., 2019). The development of hypofibrinolysis or fibrinolytic shutdown on admission does not affect prognosis, yet at 4 h following thermal injury hyperfibrinolysis as determined by TEG portends a worse prognosis (Pusateri et al., 2020).

Hemostatic profiles of patients with BIC and hyperthermia have been studied with VHAs and demonstrate superiority to CCTs for detecting BIC (Marsden et al., 2017). Additionally, VHAs have shown to be an indispensable tool for identifying the cause of hypocoagulation in patients with severe burn injury (Presnyakova et al., 2021). ROTEM was also found to be useful in providing real-time guidance for the administration of blood products in severe burns (Bugaev et al., 2020). The resuscitation of bleeding during major burn surgery has not been standardized, yet it has been noted that TEG/ROTEM analysis of intraoperative blood samples demonstrates reduced clot strength. Therefore, it has been recommended that resuscitation of patients with burn injuries in shock should aim for normal hemostasis using point-of-care VHA monitoring of hemostatic competence during surgery and resuscitation (Welling et al., 2019). Computational thrombin modeling as well as thrombin generation assays (TGA) have confirmed the validity of VHAs to guide resuscitation for patients with severe BIC who are in shock (Ball et al., 2020). The hypercoagulable state is commonly observed in post-burn patients with BIC. An etiology for this hypercoagulable state has been suggested as the hypermetabolism of fibrinogen with subsequent increase in rebound synthesis of fibrinogen. This rebound as a reaction to enhanced fibrinogen degradation manifests by increased TEG LY60 with increased clotting speed and strength by significantly increased TEG α-angle and MA (Martini et al., 2020). Recent studies of rapid TEG (rTEG) to predict resuscitation volumes and outcomes in patients with BIC have demonstrated that 75% of these patients are hypercoagulable on admission while 25% are hypocoagulable on admission. The use of VHAs to guide the complicated resuscitation of these patients has demonstrated that as much as a five-fold increase in risk of supranormal resuscitation occurs in patients with BIC and abnormally long activated clotting times. For patients with BIC and SHINE, the use of VHAs provides essential guidance for the proper utilization not only of crystalloid, but also of other blood components (Huzar et al., 2018).

An interesting therapeutic phenomenon has been reported whereby hyperthermic patients have been treated with plasma rather than crystalloids in attempt to protect the endothelial glycocalyx. Since plasma has shown to protect the glycocalyx layer of the endothelium for TIC patients, it has been proposed that plasma is a better resuscitation fluid for patients with burn wounds for its dual abilities in restoring intravascular volume and therapeutic effect on the endothelium (Gurney et al., 2019).

At the other extreme, hypothermia alters fibrinogen and platelet function as measured by VHAs. Plasma composition which is reflected by VHAs can determine the type of coagulopathy associated with hypothermia. Even moderate hypothermia can impair thrombin generation as determined by VHA analysis. Significant hypothermia demonstrates inhibition of thrombin generation as manifested by prolonged R/CT, a reduction in the α-angle, and a significant reduction of platelet function as determined by multiple electrode aggregometry (MEA) (Mitrophanov et al., 2014; Wallner et al., 2020).

### 4.7 Envenomation/intoxication

VICC uncommonly causes hemorrhagic and/or distributive shock in the western world, but much more commonly afflicts rural areas and low-to-middle income countries. However, there is also increasing frequency in urban areas in Western countries due to an interest in exotic pets. In addition to the immediate treatment with antivenom to neutralize lethal toxins, transfusion of plasma, cryoprecipitate, and specific clotting factors has been clarified by using VHAs which have demonstrated procoagulant and anticoagulant effects of snake venom. It has therefore been proposed that VHAs should goal-direct hemostatic resuscitation in patients with VICC (Park et al., 2020). Using TEG with platelet mapping (TEG/PM), a case series of rattlesnake bites in North America revealed inhibition of ADP-induced platelet activation which was reversed by Crotalidae polyvalent immune Fab (ovine). Fibrinolysis was present and resolved in patients for whom serial thromboelastographs were available (Kang and Fisher, 2020).

**FIGURE 12:**
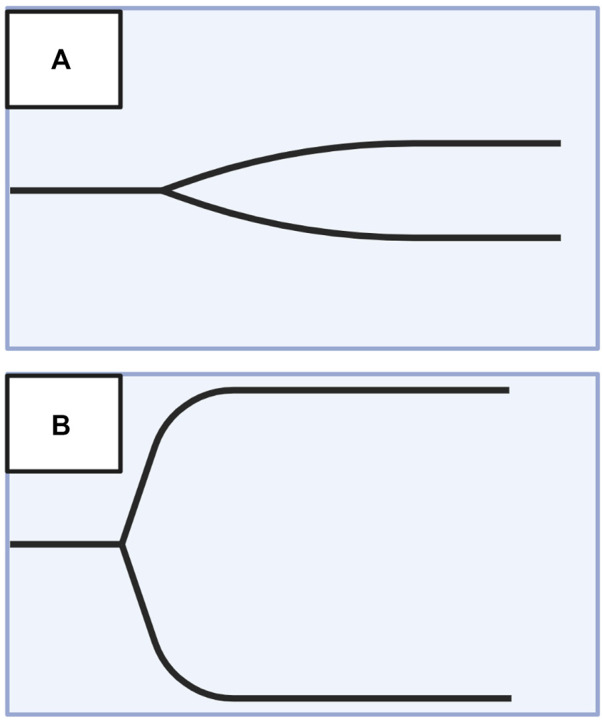
Thromboelastography (TEG) Tracings Before and After Administration of Antivenom and Blood Products. Tracing **(A)** demonstrates venom-induced consumption coagulopathy (VICC) with low α-angle and reduced maximum amplitude of a patient who required multiple rounds of antivenom to achieve hemostatic competence. Tracing **(B)** demonstrates successful treatment with resolution of VICC. In total, this patient received 24 rounds of antivenom, two units of packed red blood cells, and two units of cryoprecipitate (Leffers et al., 2018). Created with BioRender.com.

This clinical finding has been confirmed by a study comparing the efficacy of TEG and CCTs in diagnosing simulated *Crotalus* atrox envenomation using human whole blood samples. TEG accurately evaluated coagulopathies caused by *in vitro* pit viper envenomations,confirming that VHAs are useful clinical adjuncts for evaluating VICC (Leffers et al., 2018). Figure 12 demonstrates the benefit of using adjunctive VHAs as well as CCTs to diagnose and treat the coagulopathies of patients in shock caused by envenomation (Leffers et al., 2018).

### 4.8 Hematologic coagulopathies

Hematologic malignancies associate with the full spectrum of hemostatic phenotypes. Acute promyelocytic leukemia (APL) and myeloproliferative disorders such as polycythemia vera best exemplify the extremes of this spectrum. APL most often manifests a hypocoagulable hyperfibrinolytic phenotype, whereas as patients with polycythemia vera are hypercoagulable (Walsh et al., 2019; Walsh et al., 2020a). Patients with APL may present with shock associated with hyperfibrinolysis which progresses soon following treatment with all-trans-retinoic acid to a fibrinolytic shutdown phenotype (Tallman and Kwaan, 1992; Zakarija and Kwaan, 2007; Stein et al., 2009; Kwaan and Rego, 2010; Kwaan et al., 2011; Liu et al., 2011; Kwaan and Cull, 2014; Dobson et al., 2015). This pattern of rapid transition from the hyperfibrinolytic phenotype to fibrinolytic shutdown reflects a similar pattern seen in the acute phase of severe TIC which progresses from hyper-to hypo-fibrinolysis within minutes to hours (Tallman and Kwaan, 1992; Zakarija and Kwaan, 2007; Stein et al., 2009; Kwaan and Rego, 2010; Kwaan et al., 2011; Liu et al., 2011; Kwaan and Cull, 2014; Moore et al., 2015a). In APL, the hyperfibrinolytic phenotype is driven by increased expression of tPA, annexin A2, and uPA. Inhibitors of the coagulation cascade such as TFPI are present in APL and may further contribute to this hyperfibrinolytic state (Kwaan and Rego, 2010; Kwaan and Cull, 2014). Therefore, whether caused by SIC or trauma, TEG/ROTEM allow for evaluation of the patient’s phenotype along the spectrum of hypocoagulable/hyperfibrinolytic to hypercoagulable/hypofibrinolytic.

For patients with non-malignant etiologies such as hemophilias or thrombocytopenias who are in shock, VHAs may direct resuscitation by defining the hemostatic phenotype and guiding BCT and HAT (Speybroeck et al., 2020; Bunch et al., 2022a).

### 4.9 Traumatic brain injury

Coagulopathy of traumatic brain injury (CTBI) perturbs primary hemostasis, and VHAs can be used to monitor the progression of platelet dysfunction (Castellino et al., 2014; Sixta et al., 2015; Maegele et al., 2017; Samuels et al., 2019; Maegele et al., 2020; Webb et al., 2021). Early after traumatic brain injury (TBI), VHAs have shown inhibition of platelet receptors resulting in impaired reactivity at the receptors for ADP, AA (Jacoby et al., 2001; Nekludov et al., 2007; Windelv et al., 2011; Wohlauer et al., 2012; Davis et al., 2013; Castellino et al., 2014), collagen, ristocetin, and thrombin receptor activating peptides (TRAP) (Solomon et al., 2011; Kutcher et al., 2012; Dragan et al., 2021). The severity of TBI correlates to the degree of platelet receptor inhibition. Evidence suggests that CTBI may arise from the disruption of local primary hemostasis and systemic endotheliopathy which are caused primarily by the overwhelming release of von Willebrand Factor (VWF) and TF from the injured brain tissue (Wu et al., 2018; Xu et al., 2020). The circulating supraphysiologic VWF and TF likely activate platelets without adhering to a surface distant from the site of injury, creating a pool of circulating activated but exhausted platelets that are incapable of aggregation (Moore et al., 2021a). VHAs may quantify dysfunction of various platelet receptors and thus, VHAs with specialized function analysis are significantly more effective in monitoring hemostasis and prognostication in CTBI than CCTs, as has been shown for both isolated and multiple systemic trauma (Nekludov et al., 2007; Simard et al., 2009; Lustenberger et al., 2010; Kurland et al., 2012; Laroche et al., 2012; Wohlauer et al., 2012; Davis et al., 2013; Castellino et al., 2014; Nekludov et al., 2014; Hijazi et al., 2015; Medcalf, 2015; Dekker et al., 2016; Di Battista et al., 2016; Foley and Conway, 2016; Yuan et al., 2016; Maegele et al., 2017; Fletcher-Sandersjöö et al., 2020; Bradbury et al., 2021; Cannon et al., 2021). In addition to local and systemic procoagulation, patients with CTBI more commonly present with hypofibrinolysis than hyperfibrinolysis. Coupled with procoagulation, fibrinolytic shutdown leads to the consumption of platelets, coagulation factors, and fibrinogen which ultimately results in bleeding and secondary hemorrhagic progression of TBI in the late phase of CTBI (Hoffman and Monroe, 2009; Tian et al., 2010; Herbert et al., 2017; Maegele et al., 2017; Maegele et al., 2020). However, the sensitivity of VHAs to detect occult fibrinolysis in patients with CTBI is a topic of ongoing discussion (Cotton et al., 2012; Medcalf, 2015; Moore et al., 2016b; Rowell et al., 2020). Resuscitation of patients with TBI who are in shock therefore requires an understanding of the numerous contributing factors to CTBI wherein VHAs provide a greater hemostatic profile.

### 4.10 Pediatrics

Another area where definition of coagulopathies based on endothelial changes will result in much different treatments than reliance on CCTs alone is the pediatric patient whose endothelium differs significantly from the adult or PPH patient with SHINE. VHAs for decades have guided resuscitation of pediatric patients undergoing liver transplantation, cardiac surgery, cardiac transplantation, and traumatic injury. In addition, VHAs have been monitored hemostasis for patients on ECMO, VADs, continuous veno-venous hemofiltration, and continuous veno-venous plasma filtration (Haas and Faraoni, 2020). The endothelium of the pediatric population undergoes similar changes under conditions of SHINE as with the adult, although the urgency of diagnosis and treatment is much greater and therefore, consideration must be made for the use of these tests for pediatric patients in shock (Richter et al., 2022b).

Endothelial dysfunction has been implicated in pediatric critical illness and is theorized to result from glycocalyx disruption (Puchwein-Schwepcke et al., 2021). As the child develops, the endothelial glycocalyx thins due to blood vessel aging, cumulative exposure to inflammation, and other comorbidities (Richter et al., 2022b). Vascular stiffness increases with age due to physiologic elevations in blood pressure which contributes to the thinning of the glycocalyx *via* suppression of the glycocalyx core protein glypican 1 (Mahmoud et al., 2021). Additionally, the immune system develops to mount stronger responses to a wide range of pathogens, increasing baseline local and systemic inflammation in the setting of an acute response to infection (Richter et al., 2022b). Comorbid conditions such as cyanotic heart defects, lung vascular malformations, bronchopulmonary dysplasia, and type I diabetes mellitus increase endothelial exposure to reactive oxygen species which drives endothelial cells into premature senescence marked by thinning of the glycocalyx (Erusalimsky and Skene, 2009; Jackson-Weaver et al., 2019). In a critically ill pediatric patient, the joint effect of blood vessel development, immune system maturation, comorbidities, and insults such as trauma or sepsis may cause an acute disruption of the glycocalyx with subsequent vascular pathology and coagulopathies like the progression seen in SHINE. Hence, real-time monitoring of coagulopathies with VHAs may be useful in the care of critically ill pediatric patients (Saini et al., 2019).

It has been shown in pediatric intensive care units that TEG changes the treatment for 47% of patients and provides a better understanding of the hemostatic phenotype in 69% of patients (Carter et al., 2017).

The investigation of endothelial dysfunction in the pediatric population has recently been enhanced by the presence of multisystem inflammatory syndrome in children (MIS-C). Abnormal levels of angiopoietin-2, sE-selectin, and VWF antigen correlate with the vasoactive and inotropic score in patients with severe acute respiratory syndrome coronavirus 2 (SARS-CoV-2)-related MIS-C with shock (Borgel et al., 2021). For MIS-C, inflammatory markers and ROTEM parameters significantly correlate which are indicative of hypercoagulability and elevated fibrinogen activity by FIBTEM MCF and elevated D-dimer. Similar to the adult population with septic shock, pediatric patients with MIS-C who develop shock and SHINE may also benefit from the use of VHAs to guide diagnosis and resuscitation (Al-Ghafry et al., 2021).

## 5 Conclusion

The appropriate treatment of shock-associated coagulopathies mandates a personalized approach to hemostatic resuscitation. VHAs empower the clinician to appreciate the patient’s position along the spectrum of coagulofibrinolysis, and it is this appreciation of the various hemostatic phenotypes which enable the accurate diagnosis and physiologic treatment of patients with all forms of shock. Until recently, VHAs for hemostatic monitoring of patients in shock was limited to operative settings of liver transplantation and cardiac surgery. Subsequent evidenced-based protocols have validated VHA-guided resuscitation for TIC. VHAs have likewise recently expanded use to treating medical causes of bleeding associated with PPH, GI bleedings, liver failure, and septic shock. Earlier identification of coagulopathic and endotheliopathic patients with VHAs and endothelial biomarkers may enable more timely and targeted resuscitation for those patients in all forms of shock. However, further investigation is needed to elucidate the causal or correlational relationship of endotheliopathy with progressive shock, and whether restorative endothelial therapies manifest a mortality benefit.

## Glossary

AA: Arachidonic Acid
ADAM: A Disintegrin And Metalloproteinase
ADP: Adenosine Diphosphate
AFE: Amniotic Fluid Embolism
AMP: Adenosine Monophosphate
aPC: activated Protein C
APL: Acute Promyelocytic Leukemia
aPTT: activated Partial Thromboplastin Time
AT: Antithrombin III
ATP: Adenosine Triphosphate
BCT: Blood Component Therapy
BIC: Burn-Induced Coagulopathy
CCT: Conventional Coagulation Tests
CI: Coagulation Index
CTBI: Coagulopathy of Traumatic Brain Injury
DAMPs: Damage-Associated Molecular Patterns
DIC: Disseminated Intravascular Coagulation
ECMO: Extracorporeal Membrane Oxygenation
eNOS: endothelial Nitric Oxide Synthase
FDP: Fibrin/Fibrinogen Degradation Product
GAG: Glycosaminoglycan
HAT: Hemostatic Adjunct Therapy
HMGB1: High Mobility Group Box 1
HS: Heparan Sulfate
HSPG: Heparan Sulfate Proteoglycan
ICAM-1: Intercellular Adhesion Molecule-1
ISS: Injury Severity Score
LOT: Lysis Onset Time
LVAD: Left Ventricular Assist Device
MCF: Maximum Clotting Firmness
MEA: Multiple Electrode Aggregometry
MIS-C: Multisystem Inflammatory Syndrome in Children
ML: Maximum Lysis
MT: Massive Transfusion
MTH: Mild Therapeutic Hypothermia
NETs: Neutrophil Extracellular Traps
PAI-1: Plasminogen Activator Inhibitor-1
PAMPs: Pathogen-Associated Molecular Patterns
PBM: Precision-Based Medicine
PCAS: Post Cardiac Arrest Syndrome
PPH: Postartum Hemorrhage
PT: Prothrombin Time
RHAMM: Receptor of Hyaluronan-Mediated Motility
ROSC: Return Of Spontaneous Circulation
ROTEM: Rotational Thromboelastometry
rTEG: Rapid Thromboelastography
SHINE: Shock-Induced Endotheliopathy
SHOT: Systems Hypothesis of Trauma
SIC: Sepsis-Induced Coagulopathy
SOFA: Sequential Organ Failure Assessment
sTM: soluble Thrombomodulin
Syn1: Syndecan-1
Syn4: Syndecan-4
TAFI: Thrombin-Activatable Fibrinolysis Inhibitor
TBI: Traumatic Brain Injury
TEG: Thromboelastography
TF: Tissue Factor
TFPI: Tissue Factor Pathway Inhibitor
TGA: Thrombin Generation Assays
TIC: Trauma-Induced Coagulopathy
TIMP: Tissue Inhibitor of Metalloproteinase
TLR: Toll-Like Receptor
TM: Thrombomodulin
tPA: tissue Plasminogen Activator
TPE: Therapeutic Plasma Exchange
TRAP: Thrombin Receptor Activating Peptides
TXA: Tranexamic Acid
VHA: Viscoelastic Hemostatic Assay
